# Inhibitors of Serine Proteases from a *Microcystis* sp. Bloom Material Collected from Timurim Reservoir, Israel

**DOI:** 10.3390/md15120371

**Published:** 2017-12-01

**Authors:** Rawan Hasan-Amer, Shmuel Carmeli

**Affiliations:** Raymond and Beverly Sackler School of Chemistry and Faculty of Exact Sciences, Tel-Aviv University, Ramat Aviv, Tel-Aviv 69978, Israel; rawan.h8@gmail.com

**Keywords:** cyanobacteria, *Microcystis*, micropeptin, aeruginosin, protease inhibitors

## Abstract

Two new natural products, micropeptin TR1058 (**1**) and aeruginosin TR642 (**2**), were isolated from the hydrophilic extract of bloom material of *Microcystis* sp. collected from the Timurim water reservoir in Israel. The structures of compounds **1** and **2** were determined using 1D and 2D NMR spectroscopy and HR ESI MS and MS/MS techniques. Micropeptin TR1058 (**1**) was extremely unstable under the isolation conditions, and several degradation products were identified. NMR analysis of aeruginosin TR642 (**2**) revealed a mixture of eight isomers, and elucidation of its structure was challenging. Aeruginosin TR642 contains a 4,5-didehydroaraginal subunit that has not been described before. Micropeptin TR1058 (**1**) inhibited chymotrypsin with an IC_50_ of 6.78 µM, and aeruginosin TR642 (**2**) inhibited trypsin and thrombin with inhibition concentration (IC_50_) values of 3.80 and 0.85 µM, respectively. The structures and biological activities of the new compounds are discussed.

## 1. Introduction

Water bloom-forming genera of cyanobacteria, including *Microcystis*, produce biologically active natural products including toxins such as the microcystins, protease inhibitors such as the micropeptins, aeruginosins, anabaenopeptins, microginins, microviridins, and other biologically active natural products [1]. The microcystins are a group of more than 100 cyclic heptapeptides that are biosynthesized by non-ribosomal peptide synthetase. They are usually composed of 3-amino-9-methoxy-2,6,8-trimethyl-10-phenyl-4,6-decadieneoic acid (Adda), iso-linked glutamic acid, *N*Me-dehydro-alanine (Mdha) or -aminobutyric acid (Mdhb), d-alanine, a variable l-amino acid, *iso*-linked d-aspartic or methylaspartic acid, and a variable l-amino acid links to the amine of Adda [2]. The microcystins are the best characterized of cyanobacterial metabolites. These natural products have been studied for their toxicity, tumor promotion, biosynthesis [2], chemical diversity [3], and environmental impact [4].

**Figure 1:**
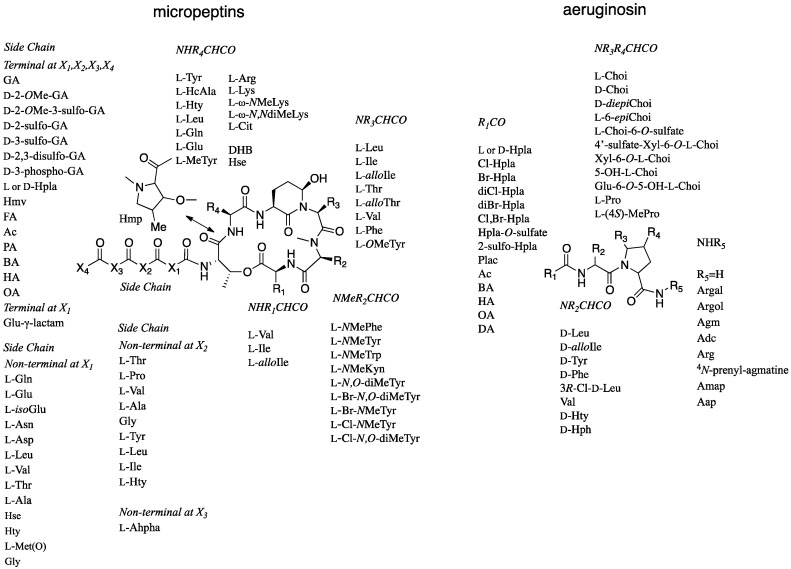
Variability of acid units within micropeptin and aeruginosin structures. Abbreviations: *N*MeKyn, *N*Me-Kynurenin; Br-*N*MeTyr, 3-Br-*N*MeTyr; Cl-*N*MeTyr, 3-Cl-*N*MeTyr; Cit, Citrulline; DHB, Dehydroaminobutiric acid; Hse, homoSerine; MeTyr, 3-MeTyr; HcAla, 3-(7-hydroxycyclohex-2-enyl)-alanyl; GA, Glyceric acid; Hmv, 2-hydroxy-3-methylvaleric acid; Hpla, p-Hydroxyphenyl lactic acid; FA, Formic acid; Ac, Acetyl; PA, Propionic acid; BA, Butyric acid; HA, Hexanoic acid; OA, Octanoic acid; isoGlu, iso-linked Glu; Hty, Homotyrosine; Ahpha, 2-amino-6-(4′-hydroxy-phenyl) hexanoic acid; Plac, Phenyllactic acid; l-Choi, (2*S*,3a*S*,6*R*,7a*S*)-2-Carboxy-6-hydroxyoctahydroindole; l-di*epi*Choi, (2*S*,3a*R*,6*R*,7a*R*)-2-Carboxy-6-hydroxyoctahydroindole; D-di*epi*Choi, (2*R*,3a*R*,6*R*,7a*R*)-2-carboxy-6-hydroxyoctahydroindole; Agm, Agmatine; Argal, Deoxyarginine; Argol, Dihydroargale; Adc, 1-(*N*-Amidino-Δ^3^-pyrrolino)-ethyl; Aap, 1-Amidino-2-amino-pyrrolidine.

Among the five types of protease inhibitors produced by cyanobacteria, the non-ribosomally synthesized micropeptins [5] and aeruginosins [6], show the greatest variability in building blocks. This variability leads to numerous analogs that inhibit serine proteases with a comparable potency (Figure 1). The micropeptins, with at least 153 characterized members [7], compose the largest group of these protease inhibitors. The aeruginosins, with at least 72 members [8], are second in the number of analogs. The micropeptins are cyclic depsipeptides composed of a ring of six amino acids and a side chain of one to four amino acid units. The 19-membered ring contains a unique 3-amino-6-hydroxypiperidinone (Ahp) moiety and an α-amino-β-hydroxy acid (l-threonine or β-hydroxy-γ-methylproline). The β-hydroxy-group of the latter amino acid forms a lactone ring with the amino acid at the carboxylic terminus of the peptide, while the side chain is anchored to the ring through its α-amino group (Figure 1).

Whether micropeptins inhibit trypsin or chymotrypsin types of serine proteases is primarily determined by the nature of the amino acid connected to the *N*-terminus of the Ahp. Lipophilic amino acids, such as glutamine (Gln), leucine (Leu), homotyrosine (Hty), dehydroaminobutiric acid (Dhb), and 3-(7-hydroxycyclohex-2-enyl)-alanyl (HcAla), attached to the *N*-terminus of Ahp, confer selectivity for inhibition of chymotrypsin, whereas basic amino acids, such as lysine (Lys) and arginine (Arg), result in the inhibition of trypsin-related serine proteases [9]. The aeruginosins (Figure 1) are composed of three or four building blocks, a hydroxyl acid or a fatty acid at the *N*-terminus, a lipophilic amino acid at the second position, 2-carboxy-6-hydroxyoctahydroindole (Choi) or proline at the third position, and, if present, an arginine derivative at the fourth position (the “carboxylic terminus”) of the modified peptide [6]. The aeruginosins inhibit trypsin-type serine proteases, but only those that contain the arginine derived unit are active [6].

The chemical diversity of these secondary metabolites in bloom material is usually high and can be easily monitored by MALDI TOF MS analysis of small amounts of bloom material [10]. MALDI TOF MS analysis of samples of cyanobacterial bloom materials collected few meters apart from one another are usually highly chemical diverse although under a microscope and genetically the bloom may appear homogenous [11]. As part of our ongoing research on the chemistry and chemical ecology of cyanobacterial blooms in water bodies [7], a biomass of a *Microcystis* sp. bloom material (TAU collection No. IL-428), collected from a secondary water pond in Timurim, Israel, on 14 August 2013, was studied. Two new protease inhibitors, micropeptide TR1058 (**1**) and aeruginosin TR642 (**2**), were identified in this sample. The structure elucidation and biological activity of **1** and **2** are discussed below.

## 2. Results and Discussion

**Figure 2:**
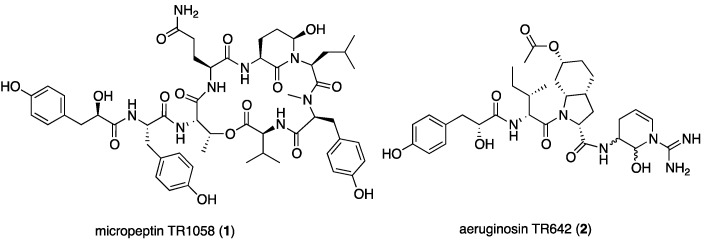
Structures of the compounds isolated from *Microcystis* sp. IL-428.

The aqueous methanol (7:3) extract of the bloom material afforded seven related micropeptins and one aeruginosin, but did not contain any microcystin derivatives. Six of the seven micropeptins were isolation artifacts of micropeptin TR1058 (**1**), which was extremely unstable under the isolation conditions and afforded the six degradation products derived from trifluoroacetic acid (TFA) catalyzed hydrolysis of the glutamine and Ahp residues (see products in Figure S1 and NMR data in Tables S1 and S2 in the Supplementary Materials). This phenomenon was confirmed by LC-MS of the crude extract. Peaks corresponding to only two peptides, micropeptide TR1058 (**1**) and aeruginosin TR642 (**2**), were observed (Figure 2).

### 2.1. Structural Elucidation of Micropeptin TR1058

Micropeptin TR1058 (**1**) was isolated as an amorphous white powder possessing a negative high-resolution ESI-MS molecular ion at *m*/*z* 1057.4882 ([M − H]^−^), corresponding to the molecular formula of C_53_H_70_N_8_O_15_ and 24 degrees of unsaturation. The 1D and 2D NMR of **1** (Table 1) were measured in DMSO-*d*_6_. The ^1^H NMR spectrum of **1** revealed its peptide nature (i.e., five secondary doublet amide protons (δ_H_ 8.50–7.40), two singlet amide protons (δ_H_ 7.19 and 6.74) and a singlet amide methyl group). Furthermore, the spectrum revealed three singlet phenol and twelve doublet aromatic protons of three para-substituted phenol rings (δ_H_ 9.20, 9.11 and 9.07, singlets 1H each and 6.97, 6.92, 6.88, 6.62, 6.61 and 6.60, doublets 2H each), and two downfield shifted oxymethine protons (δ_H_ 5.49, q and 4.88, brs). The ^13^C NMR spectrum of **1** revealed two oxymethine carbons (δ_C_ 72.1 and 73.6) (Table 1). The later oxymethine signals together with the singlet amide methyl group are characteristic of micropeptin type depsipeptides [9].

**Figure 3:**
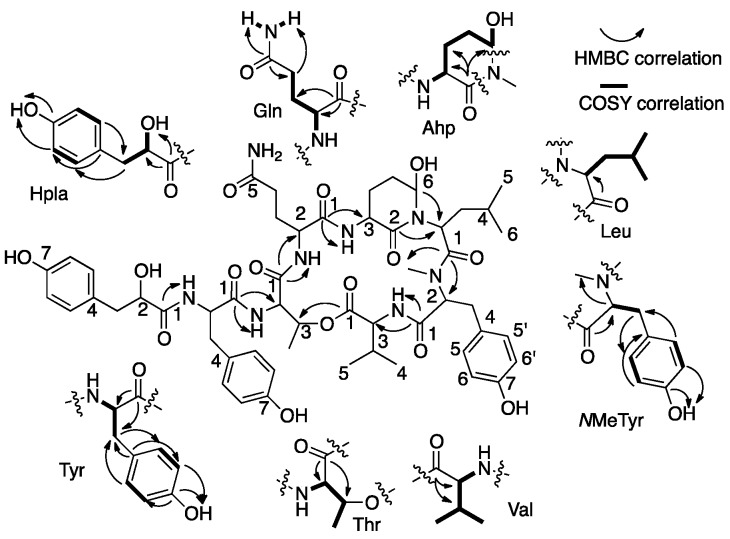
COSY and HMBC correlations that supported the structure elucidation of **1**.

Analyses of the 1D (^1^H and ^13^C) and 2D (HSQC, HMBC, COSY, and TOCSY) NMR data allowed the assignment of eight substructures (Figure 3). COSY and TOCSY correlations (bold lines in Figure 3) established the connectivity of the protons from the α-NH or α-OH through the consecutive proton spin system of the side-chain of the residue and of other isolated spin systems. HSQC correlations (Table 1) assigned the carbons to the later protons. HMBC correlations (Figure 3, curved arrows, and Table 1) connected the later spin systems through quaternary and carbonyl carbons to enable identification of the eight substructures: amino hydroxyl piperidone (Ahp), glutamine, *N,N*-disubstituted leucine, *O*-substituted threonine, tyrosine, *N*Me-tyrosine, valine, and the hydroxy-acid, p-hydroxyphenyllactic acid (Hpla) (Figure 3 and Table 1). The assembly of the amino acids into the planar structure of **1** was deduced from the HMBC correlations (Figure 3), observed from the carbonyl of one amino acid to the α-CH and NH or *N*Me of the neighboring amino acid residues. The structure could also be fully assembled by nuclear Overhauser effect (NOE) correlations from the ROESY experiment (Table 1).

The 3S*,6R* relative configuration of Ahp was established by comparison of its ^1^H and ^13^C NMR data with those of micropeptin LH911A [12], the NOE of Ahp-NH with the pseudoaxial H-4b (δ_H_ 2.54), and the NOE of the latter with the pseudoaxial 6-OH. Acid hydrolysis of **1** and derivatization with Marfey’s reagent [13], followed by HPLC analysis, demonstrated the L configurations of valine, *N*Me-tyrosine, leucine, glutamic acid, threonine, and tyrosine residues. Marfey’s analysis using 1-fluoro-2,4-dinitrophenyl-5-l-alanine amide (FDAA) as Marfey’s reagent [13] fails to distinguish l-threonine from l-*allo*-threonine, and thus additional evidence was needed to support the absolute configuration of the *N,O*-disubstituted-threonine. A comparison of the *J*-value between H-2 and H-3, in all known micropeptins (0–1 Hz) [14], with the one observed for 1 (~1 Hz, broadening of the signal) revealed the (2*S*,3*R*)-absolute configuration of the latter l-threonine. To establish the absolute configuration of C-3 of the Ahp moiety, 1 was oxidized using the Jones reagent [15] to produce the corresponding 3-aminopiperidine-2,6-dione from the Ahp residue. The oxidation was followed by a hydrolysis, derivatization, and Marfey’s analysis to determine the 3*S*-configuration for the Ahp residue (hydrolysis liberated l-glutamic acid from Ahp). The configuration of C-6 of the Ahp moiety was thus determined as *R*. The absolute, *S* (l)*,* configuration of C-2 of Hpla was established by comparison of its retention time on a chiral HPLC column with the retention times of authentic d-and l-Hpla. Based on the arguments described above, the structure of micropeptin TR1058 was established as 1 (Figure 2).

### 2.2. Structural Elucidation of Aeruginosin TR642

Aeruginosin TR642 (**2**) was isolated as a glassy material that possessed a positive HR-ESI-MS adduct ion at *m*/*z* 643.3412 ([M + H]^+^), corresponding to a molecular formula of C_32_H_46_N_6_O_8_ and 13 degrees of unsaturation. Despite the moderate size of **2**, which was inferred from its mass weight, the ^1^H and ^13^C NMR spectra in DMSO-*d*_6_ (and in other deuterated NMR solvents) were extremely complicated. The 1D NMR spectra of **2** presented many signals corresponding to amide protons (δ_H_ 8.40–7.30), amine-bearing carbons (δ_C_ 60.0–45.0), and carbons that could be attributed to amide carbonyls (δ_C_ 173.5–169.0), all pointing to a peptide nature. Heating the solution of **2** in DMSO-*d*_6_ to 340 °K resulted in a better-resolved NMR spectrum, but the complexity remained higher than expected from its molecular weight. Careful examination of the ^13^C NMR spectrum in DMSO-*d*_6_ revealed that, at room temperature, several peaks appeared as eight-line multiplets (four at about same height and the other four at about half height); others appeared broad, and few were sharp signals. In contrast, at 340 °K the eight-line multiplets collapsed to four-line multiplets, and the other signals were sharpened. Our interpretation of this behavior is that one of the doublings results from restricted rotation, while the other two are due to four diastereoisomers of two chiral centers.

**Figure 4:**
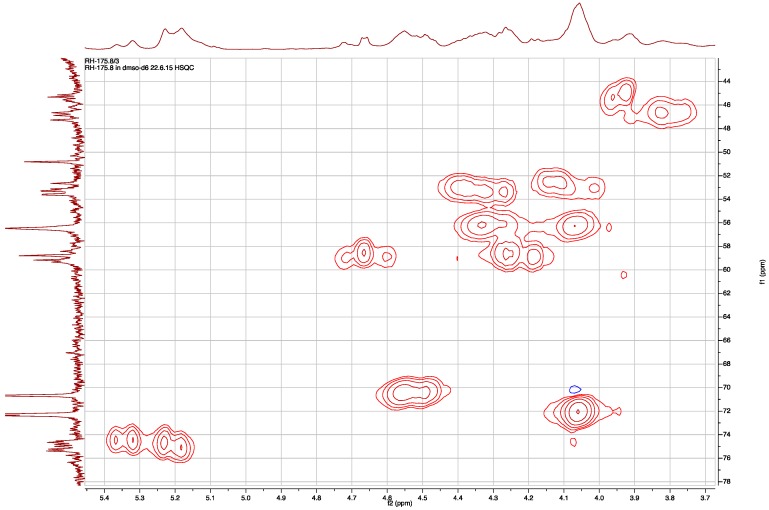
Expansion of the HSQC map of **2** showing the distribution of the proton and carbon multiplets in both dimensions.

The HSQC spectrum in DMSO-*d*_6_ at room temperature revealed that the wide multiplets in the carbon dimension were in the region of 110 to 22 ppm, and the carbons centered at 46.5 and 75.0 ppm presented the widest multiplets (Figure 4). Combining the information from ^1^H, ^13^C, HSQC, and COSY spectra, an acetate group (3H, two signals δ_H_ 1.98 and 1.94, in 5:3 ratio), a para-substituted phenol ring (δ_H_ 9.12 brs, 1H; 7.00 and 6.98 two overlapping doublets, 2H; 6.62 two overlapping doublets, 2H), a benzylic methylene coupled to hydroxymethine (δ_H_ 2.88 m, 1H; 2.61 m, 1H; 4.06 brs, 1H; 5.70–5.49 br, 1H), an isobutyl moiety (δ_H_ 0.71–0.73, 0.81 t, 3H; δ_C_ 11.68–12.14, CH3; δ_H_ 1.19, 1.10, m, 1H; δ_C_ 26.63–26.75, CH_2_; δ_H_ 0.93, 0.83, m, 1H, 1.64, 1.59, m, 1H; δ_C_ 34.96–35.67 and 37.01–37.42, CH; δ_H_ 0.73, 0.69, d, 3H; δ_C_ 13.52–13.70 and 14.16–14.29 CH_3_), a guanidine moiety (δ_H_ 7.90, brs 3H; δ_C_ 154.70–155.68, C), and four methine signals characteristic of a Choi moiety (δ_H_ 4.18–4.28 and 4.50–4.71, d; δ_C_ 58.42–58.95 and 59.16–59.76, CH; δ_H_ 2.25–2.39 and 2.40–2.51, m; δ_C_ 31.99–32.33 and 34.64–34.75, CH; δ_H_ 4.49, brt, and 4.55, m, 1H; δ_C_ 70.65, CH; δ_H_ 4.06, m and 4.34, m 1H; δ_C_ 56.47 and 56.55, CH), all pointed to the conclusion that 2 is an aeruginosin-type linear peptide. This explained the collapsing of one of the doublet in the ^1^H and ^13^C NMR spectra when the sample was heated to 340 °K. The doublet resulted from restricted rotation around the amide bond between the carbonyl of the amino acid at position 2 and tertiary nitrogen of the Choi at position 3.

The elucidation of the structures of the four subunits was achieved in a similar way to that of **1**. The Hpla unit was assembled based on the COSY correlations of H-5,5′ with H-6,6′ and H-2 with 2-OH, H-3a, and H-3b. Carbons bearing these protons were assigned based on HSQC correlations. Final assembly of the quaternary carbons of this unit into the full structure was accomplished based on HMBC correlations (Table 2, 7-OH with C-6,6′,7; H-6,6′ with C-4; H-5,5′ with C-3 and C-2; H-3a and 3b with C-4 and C-1). The isobutyl residue (proposed based on COSY and HSQC correlations) was further connected to an amino methine through COSY and HSQC correlations and to an amide carboxyl carbon through the HMBC correlations of the eight resonances of the later amino methine (Ile-H-2) with the resonances of the carboxyl (δ_C_ 169.2-169.7). COSY and TOCSY correlations allowed the construction of the proton spin system from Choi-H-2 through H-3eq, H-3ax, H-3a, H-7a, H-7eq, H-7ax, H-6, H-5eq, and H-5ax to H-4eq and H-4ax but failed to connect unequivocally H-3a with H-4eq and H-4ax. HSQC correlations assigned the carbon signals to the protons in the sequence (Table 2). HMBC correlations supported the assignment of the sequence of proton and carbon signals, including the connection of the *O*-acetyl moiety to C-6, but again failed to unequivocally support the connection of methine-3a to methylene-4. Comparison of the carbon and proton chemical shifts of the Choi moiety of **2** with those of (2*R*,3a*R*,6*R*,7a*R*)-Choi-6-*O*Ac in aeruginosin GH553 [15], (2*S*,3a*S*,6*R*,7a*S*)-Choi in aeruginosin KY642 [16], and (2*S*,3a*S*,6*S*,7a*S*)-Choi in aeruginosin DA495 [17] revealed that 2 contains either (2*R*,3a*R*,6*R*,7a*R*)-Choi-6-*O*Ac or (2*S*,3a*S*,6*S*,7a*S*)-Choi-6-*O*Ac.

The carbon and proton signals of the last subunit of **2** presented the widest multiplets of all four subunits. Its structure elucidation was complicated by the overlap of multiple signals from Ddha-1-OH and Ddha-5-H. Understanding that the latter two signals overlapped in the proton dimension facilitated the elucidation of the structure of the didehydroaraginal (Ddha) moiety. COSY correlations established the proton spin system from 1-OH through H-1, H-2, 2-NH, H-3a, H-3b, H-4, and H-5, and HSQC correlations assigned the carbon chemical shifts of the corresponding carbons. The chemical shift of C-1 (δ_C_ 74.52–75.76, similar to Ahp-C-6 in 1) and C-4 and C-5 (δ_C_ 107.38–108.86 and 121.45–122.19, respectively) suggested that both C-1 and C-5 are connected to a nitrogen. The mutual correlations of H-1 with C-5 and H-5 with C-1 suggest that C-1 and C-5 are connected through the same nitrogen indicative of a trisubstituted tetrahydropyridine moiety. The last substituent connected to the latter nitrogen was assigned through the HMBC correlation of H-5 with the guanidine carbon (δ_C_ 154.70–155.68), although the amine protons (δ_H_ 7.90) assigned to it did not show an HMBC correlation with it. Based on this analysis, the structure of the subunit was assigned as a mixture of the four diastereoisomers of cyclic didehydroaraginal, explaining two of the three doublet NMR signals mentioned above. The cyclic didehydroaraginal is described here for the first time, but diasteromeric cyclic araginal moieties were described in the past as part of several aeruginosins (i.e., aeruginosins 89-A and -B [18], 102-A and -B [19], 103-A [20], 686A and B [21], and LH650A and B [12]).

**Figure 5:**
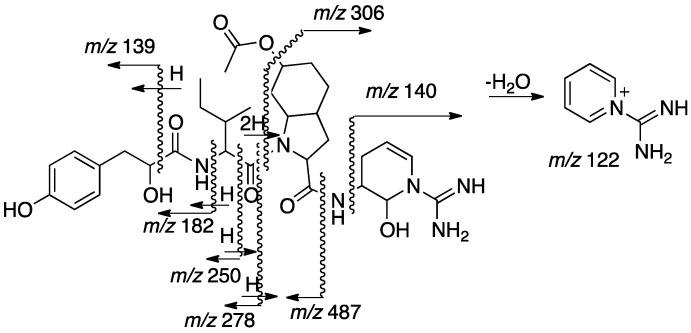
Sequence assignment of **2** based on fragmentation ions from the collision induced decomposition (CID) ESI-MS molecular ion.

The sequence of the subunits that compose 2, Hpla-Ile-Choi(6-OAc)-Ddha, was established based on HMBC correlations between Ile-H-2 and Ile-NH and Hpla-CO, Choi-H-2 and Ile-CO, and Ddha-2-NH and Choi-CO. The planar structure of **2** was supported by its CID ESI MS/MS fragmentation pattern (Figure 5). The molecular formula calculated for the proposed structure, C_32_H_46_N_6_O_8_, matched the one established by the HR ESI MS. The absolute configuration of the chiral centers of the isoleucine moiety were assigned by Marfey’s analysis [13] as d-*allo*Ile (2*R*,3*S*), those of the Choi were established by advanced Marfey’s method [22] to be (2*R*,3a*R*,6*R*,7a*R*)-Choi, and that of the hydroxyl-acid was established by chiral HPLC to be d-Hpla. The d-*allo*Ile absolute configuration was supported by the chemical shifts of Ile-C-5 and C-6 (δ_C_ centered at 11.9 and 13.9), which matched those of d-*allo*Ile in aeruginosins 98-C and 101 (δ_C_ 11.6 and 13.8 and, 11.8 and 13.6, respectively) [18] and l-*allo*Ile in oscillamide J (δ_C_ 11.4 and 14.3) [23], but differed from l-Ile in nostopeptin A (δ_C_ 11.3 and 16.1) [24]. Based on these arguments, structure 2 was assigned to aeruginosin TR642 (Figure 2).

The micropeptins and the aeruginosins are biosynthesized by non-ribosomal peptide synthetases that are flexible and capable of synthesizing analogues metabolites [21,25]. Aeruginosin TR642 contains a 4,5-didehydroaraginal subunit that had not described before. Biosynthetically, 4,5-didehydroaraginal is probably produced from arginine via dehydroarginine. Dehydroarginine was recently shown to be synthesized in *Streptomyces griseus* from arginine by pyridoxal phosphate-dependent enzymes [26]. A similar enzyme system is probably responsible for the synthesis of Ddha in the cyanobacterium with reductive release of the tetrapeptide from the enzyme [27].

### 2.3. Biological Activity Assessment

The biological activities of **1** and **2** were examined against serine proteases. Micropeptin TR1058 (**1**) inhibited chymotrypsin with an IC_50_ value of 6.78 µM, but did not inhibit elastase, trypsin, or thrombin. Aeruginosin TR642 (**2**) inhibited trypsin and thrombin with IC_50_ values of 3.80 and 0.85 μM, respectively. The activity of micropeptin TR1058 (**1**) is in line with the structure-activity relationship of the Ahp-containing cyclic micropeptins [9]. Structurally related micropeptin KR1030 inhibits chymotrypsin and elastase with IC_50_ values of 13.9 and 28.0 μM, respectively [28]. Interestingly, the acid-catalyzed hydrolysis of the glutamine residue of **1** in the acidic conditions of the HPLC mobile-phase (0.1% TFA/MeCN) produced glutamic acid and methoxyglutamate, and neither inhibited chymotrypsin catalytic activity.

Aeruginosin TR642 (**2**) was active against both trypsin and thrombin as expected based on activities of aeruginosins that contain arginine-derived residues at the carboxylic end of the modified peptide [6]. Aeruginosins LH650A, LH650B, and LH606 inhibit trypsin with IC_50_ values of 37.9, 35.3 and 18.5 µM, respectively, and thrombin with IC_50_ values of 1.8, 1.8 and 2.5 µM, respectively [8]. This finding adds a new derivative, 4,5-didehydroaraginal (Ddha), to the known building blocks of trypsin-type serine protease inhibiting aeruginosins; others are agmatine, araginol, araginal, 3-aminoethyl-1-*N*-amidino-Δ^3^-pyrroline [3] and 1-amidino-2-aminopyrrolidine (Aap) [12]. A recent study showed that aeruginosin K-139, an araginal containing aeruginosin, blocks the formation of the activated factor VII-soluble Tissue Factor complex (fVIIa-sTF, EC_50_ ~166 µM) and inhibits thrombin catalytic activity (EC_50_ of 0.66 µM) [29]. It will be interesting to compare the fVIIa-sTF activity of aeruginosin TR642 with that of aeruginosin K-139 in view of the opposite absolute configuration of the Ile/Leu and Choi sub-units and Ddha versus araginal, respectively. Unfortunately, due to insufficient amounts of **2**, we were unable to perform this experiment.

## 3. Materials and Methods

### 3.1. General Experimental Procedures

Optical rotation values were obtained on a Jasco P-1010 polarimeter (Jasco, Oklahoma City, OK, USA) at the sodium D line (589 nm). UV spectra were recorded on an Agilent 8453 spectrophotometer (Santa Clara, CA, USA). IR spectra were recorded on a Bruker Tensor 27 FT-IR instrument (Billerica, MA, USA). NMR spectra were recorded on a Bruker Avance and Avance III spectrometers (Billerica, MA, USA) at 500.13 MHz for ^1^H and 125.76 MHz for ^13^C. DEPT, COSY-45, gTOCSY, gROESY, gHSQC, gHMQC, and gHMBC spectra were recorded using standard Bruker pulse sequences. NMR chemical shifts were referenced to TMS δ_H_ and δ_C_ = 0 ppm. High-resolution mass spectra were recorded on a Waters MaldiSynapt instrument (Waters, Milford, MA, USA) (ESI), and LC-MS spectra were recorded on a Waters Xevo TQD instrument (Waters, Milford, MA, USA) (ESI). HPLC separations were performed on a Merck Hitachi HPLC system (l-6200 Intelligent pump and l-4200 UV-VIS detector), a JASCO P4-2080 plus HPLC system with a Multiwavelength detector, and an Agilent 1100 Series HPLC system.

### 3.2. Biological Material

*Microcystis* sp. (collection No. IL-428) was collected from a secondary water pond (No. 3) in Timurim, Israel, on 14 August 2013. The sample was identified by microscopic observation as a *Microcystis* sp. A lyophilized voucher sample (IL-428) was deposited in the culture collection of Tel Aviv University (Tel Aviv, Israel).

### 3.3. Isolation Procedure

The cell mass was separated from the aqueous solution, frozen, and lyophilized. The freeze-dried cells (IL-428, 493 g) were extracted with 7:3 MeOH:H_2_O (3 × 3 L). The crude extract (415 g) was evaporated to dryness. Fatty acids and salts were removed from the crude extract with petroleum ether and methanol, respectively. Aliquots of the extract were fractionated (10 g in each separation) on an (ODS, YMC-GEL, 120A, 4.4 × 6.4 cm) reversed-phase flash column with an increasing concentration of MeOH in H_2_O. The combined fraction 7 (6:4 MeOH:H_2_O, 3.03 g) was subjected to a Sephadex LH-20 column (the fraction was divided into three equal portions that were loaded onto the column separately) in 1:1 CHCl_3_:MeOH to obtain 17 fractions. Fractions 5 + 6, 8–13, and 8–10 from the three column separations were combined based on their NMR spectra (803.5 mg) and were resolved over a Sephadex LH-20 column (1:1 CHCl_3_:MeOH) to obtain 12 fractions. Fractions 4–6 from the Sephadex LH-20 column were further separated on a Sephadex LH-20 column in 7:3 MeOH:H_2_O to obtain seven fractions. Fraction 3 (98.4 mg) was separated on a reversed-phase HPLC (YMC C-18, 10 μm, 250 × 20 mm, diode array detector (DAD) at 238 nm, flow rate 5.0 mL/min) in 55:45 0.1% aq. TFA:MeCN to obtain seven fractions. Fraction 3 (58.1 mg) was further separated on a reversed-phase HPLC (YMC C-18 Hydro RP, 10 μm, 250 × 21.2 mm) in 58:42 0.1% aq. TFA:MeCN to obtain six fractions. Fraction 1 (10.6 mg) was found to be pure and was named micropeptin TR1058 (**1**) (4.5 mg, *R*_t_ 15.27 min, 9.1 × 10^−4^% yield based on the dry weight of the cells). Pure fractions 2 and 3 were named micropeptins TR1059 (6, 2.3 mg, *R*_t_ 18.17 min, 4.6 × 10^−4^% yield based on the dry weight of the cells) and micropeptin TR1072 (3, 26.2 mg, *R*_t_ 23.63 min, 4.7 × 10^−3^% yield based on the dry weight of the cells), respectively. Fractions 7–10 from the second Sephadex LH-20 column were further separated on a reversed-phase HPLC (YMC C-18 Hydro RP, 10 μm, 250 × 21.2 mm) in 57:43 0.1% aq. TFA:MeCN to obtain nine fractions. Fraction 1 was further separated over the same column in 7:3 MeOH:H_2_O to obtain five fractions. Pure fraction 1 was named micropeptin TR1073a (4, 1.1 mg, *R*_t_ 15.1 min, 2.2 × 10^−4^% yield based on the dry weight of the cells), fraction 6 was found to be a mixture of two micropeptins, micropeptins TR1073b (7) and TR1090 (8, 7.1 mg, *R*_t_ 27.12 min, 1.4 × 10^−3^% yield based on the dry weight of the cells), and pure fraction 7 was named micropeptin TR1087 (5, 23.9 mg, R_t_ 38.5 min, 4.8 × 10^−3^% yield based on the dry weight of the cells). The combined fraction 9 from the initial reversed-phase separation (8:2 MeOH:H_2_O, 999.9 mg) was subjected to a Sephadex LH-20 column in 1:1 MeOH:CHCl_3_ to obtain twenty fractions. Fractions 8–14 (400 mg) were separated on a reversed-phase HPLC (YMC C-18 Hydro RP, 10 μm, 250 × 21.2 mm) in 61:39 0.1% aq. TFA:MeCN to obtain five fractions. Fraction 3 (44.6 mg) was subjected to a Sephadex LH-20 1:1 CHCl_3_:MeOH to give 11 fractions. Fractions 5–10 were combined and separated on reversed-phase HPLC on the same column in 7:3 0.1% aq. TFA:MeCN to obtain aeruginosin TR642 (**2**) (13.2 mg, *R*_t_ 13.37 min, 2.6 × 10^−3^% yield based on the dry weight of the cells).

Micropeptin TR1058 (**1**): amorphous white powder; ${\left[\alpha \right]}_{D}^{20}$ −5.7 (*c* 0.28, MeOH); UV (MeOH) *λ*_max_ (log ε) 201 (4.32), 225 (3.91), 278 (3.13) nm; IR (ATR Diamond): 3294, 2360, 2341, 1733, 1517 cm^−1^; for NMR data see Table 1; HR ESI MS *m*/*z* 1057.4875 ([M − H]^−^; calc. for C_53_H_69_N_8_O_15_, 1057.4882).

Aeruginosin TR642 (**2**): glassy material; ${\left[\alpha \right]}_{D}^{20}$ 4.4 (*c* 0.63, MeOH); UV (MeOH) *λ*_max_ (log ε) 201 (4.08), 223 (3.82) nm; IR (ATR Diamond): 3273, 2360, 2341, 1670, 1558 cm^−1^; for NMR data see Table 2; HR ESI MS *m*/*z* 643.3450 ([M + H]^+^; calc. for C_32_H_47_N_6_O_8_, 643.3455).

### 3.4. Determination of the Absolute Configuration of the Amino Acids by Marfey’s Method

In the case of **1**, one sample (0.5 mg in 1mL of acetone at 0 °C) was oxidized with Jones reagent prior to the hydrolysis. Compounds **1**, oxidized-**1**, and **2** (0.5 mg each) were hydrolyzed in 6 N HCl (1 mL) and analyzed by Marfey’s method [13]. The reaction mixture was maintained in a sealed glass bomb at 104 °C for 18 h. The acid was removed *in vacuo*, and the residue was re-suspended in 250 μL of H_2_O. FDAA solution (1-fluoro-2,4-dinitrophenyl)-5-l-alanine amide in acetone (0.03 M, 20 μL per each amino acid in the peptide) and NaHCO_3_ (1 M, 20 μL per each amino acid) were added to each reaction vessel. The reaction mixture was stirred at 40 °C for 2.5 h in the dark. HCl (2 M, 10 μL per each amino acid) was added to each reaction vessel, and the solution was evaporated in vacuo. The FDAA-amino acids derivatives from hydrolysate were dissolved in 1 mL CH_3_CN and compared with standard FDAA-amino acids by an HPLC analysis: LiChroCART RP-18 column (5 μm, 250 × 4.6 mm); flow rate 1 mL/min; ultraviolet (UV) detection at 340 nm; linear gradient elution from 0.1% aq. TFA buffer (pH 3) to 6:4 MeCN:0.1% aq. TFA buffer (pH 3) within 60 min. The absolute configuration of each amino acid was determined by spiking the derivatized hydrolysates with a d,l-mixture of the standard derivatized amino acids.

Compound **2** was also analyzed by the advanced Marfey method [19]. Two 0.5 mg portions were hydrolyzed as described above and derivatized, one with l-FDAA and the other with d-FDAA. The two samples of l- and d-FDAA derivatives were analyzed by ESI LC MS. The analysis was performed on a Waters Acquity UPLC coupled with a UV detector (Waters Acquity-TUV detector) (Waters, Milford, MA, USA) and mass spectrometer (Waters Xevo TQD) (Waters, Milford, MA, USA) on a C18 (1.7 μm, 2.1 Å, ~100 mm) column (Waters Milford, MA, USA) The mobile phase compositions were (A) 95:5 H_2_O/MeCN + 0.1% formic acid (FA) and (B) MeCN + 0.1% FA. The elution gradient was as follows: 1 min of 100% A, linear gradient to 40% B over 25 min, and hold for 4 min. Samples of 10 μL were injected, and the flow rate was 0.5 mL/min. The UV detector was set to 340 nm, and the mass spectrometer was operated in both negative and positive ion modes, scanning between 200 and 650 mass units. The interpretation of the data was conducted after the run on both positive and negative ion modes by MassLynx software (v4.1, Waters Laboratory Informatics).

### 3.5. Determination of the Absolute Configuration of Hydroxy Phenyl Lactic Acid (Hpla)

Hpla was extracted from the acid hydrolysates of compounds **1** and **2** with ethyl ether. The ether was removed in vacuo, and the residue was dissolved in triethylammonium acetate (15 mM) in MeOH (0.5 mL). The MeOH solution was analyzed on an Astec, Chirobiotic T, LC stationary phase, 250 × 4.6 mm (5 μm), flow rate 1 mL/min, UV detection at 277 nm, linear elution with 19:1 MeOH:1% aq. triethylammonium acetate buffer (pH 4). The retention times of the Hpla derivatives from **1** and **2** were compared with an authentic standard of d,l-Hpla under the same conditions.

### 3.6. Protease Inhibition Assays

The samples for biological assays were dissolved in DMSO at a concentration of 1 mg/mL, and the IC_50_ values were determined by analysis of a series of dilutions (from 0.011 µM to 45.5 µM). A sigmoidal curve of the enzyme inhibition versus the concentration of the inhibitor was observed that was fit to the 4-parameters logistic model [30]. Assays were performed in 96-well plate format.

#### 3.6.1. Trypsin

The assay was performed in a Tris buffer (0.6 g TRIS HCl/100 mL H_2_O, pH 7.5). Benzoyl-l-arginine-*p*-nitroanilide hydrochloride (BAPNA), the trypsin substrate, was dissolved at 1 mg/mL in 1:9 DMSO/buffer. The enzyme was dissolved in buffer at 1 mg/mL. To each well were added 100 µL of buffer, 10 µL of enzyme, and 10 µL of sample. The plate was placed in the spectrophotometer at 37 ° C. After 5 min, 100 µL of substrate solution was added to each well, and the plate was placed in the spectrophotometer for the kinetic measurement of the absorbance intensity over 30 min at a wavelength of 405 nm.

#### 3.6.2. Thrombin

The assay was performed in a Tris buffer (2.422 g TRIS HCl/100 mL H_2_O, pH 8.0). Z-Gly-Pro-Arg-4MβNA acetate salt, the thrombin substrate, and the enzyme, were dissolved (separately) the buffer at concentrations of 0.5 mg/mL. To each well were added 170 µL of buffer solution, 10 µL of enzyme, and 10 µL of sample. The plate was placed in the spectrophotometer for at 25 °C. After 20 min, 30 µL of substrate solution was added to each well, and the plate was replaced in the spectrophotometer for the kinetic measurement of the absorbance intensity over 20 min at a wavelength of 405 nm.

#### 3.6.3. Chymotrypsin

The assay was performed in a Tris buffer (0.6 g TRIS HCl/100 mL H_2_O, pH 7.5). The enzyme and the substrate Suc-Gly-Gly-phenylalanine-*p*-nitroanilide (SGGPNA) were dissolved in the buffer at concentrations of 1 mg/mL. To each well were added 100 µL of buffer, 10 µL of enzyme, and 10 µL of sample. The plate was placed in the spectrophotometer at 37 °C. After 5 min, 100 µL of substrate solution was added to each well, and the plate was placed in the spectrophotometer for the kinetic measurement of the absorbance intensity over 30 min at a wavelength of 405 nm.

#### 3.6.4. Elastase

The assay was performed in a Tris buffer (2.422 g TRIS HCl/100 mL H_2_O, pH 8.0). The enzyme and the substrate, *N*-Suc-Ala-Ala-Ala-*p*-nitroanilide, were dissolved in the buffer at a concentration of 75 mg/mL and 0.9 mg/mL, respectively. To each well were added 150 µL of buffer solution, 10 µL of enzyme, and 10 µL of sample were added to each well. The plate was placed in the spectrophotometer at 30 °C. After 20 min, 30 µL of substrate solution was added to each well, and the plate was replaced in the spectrophotometer for the kinetic measurement of the absorbance intensity over 20 min at a wavelength of 405 nm.

## 4. Conclusions

*Microcystis* blooms usually contain various species or chemotypes that produce a large variety of short peptides; the micropeptins are dominant in quantity and diversity [10]. The toxic microcystins are second in diversity and are usually present in minute quantities relative to the micropeptins [2]. In the current research, the 7:3 MeOH:H_2_O extract of a *Microcystis* sp. bloom material, which seemed homogenous under the microscope, yielded only two modified peptides of two structural groups, the micropeptins and the aeruginosins, but none of the toxic microcystins. Our structural analyses demonstrated that aeruginosin TR642 (**2**) contains a 4,5-didehydroaraginal subunit that had not been described before. The biosynthesis of Ddha in this *Microcystis* strain confirmed the high versatility of the biosynthetic machinery of *Microcystis* spp. in particular and cyanobacteria at large [31,32]. The reasons for the biosynthesis of these metabolites and their high variability in cyanobacterial water blooms remain unclear, and our current research is aimed at revealing the purpose for the biosynthesis of these metabolites.

## Figures and Tables

**Table 1:** NMR data of micropeptin TR1058 (**1**) in DMSO-*d*_6_^a^.

Position	δ_C,_ Type ^b^	δ_H_, mult., *J* (Hz)	LR CH Correlations ^c^	NOEs ^d^
Val	-	-	-	-
1	172.5, C	-	-	-
2	56.2, CH	4.72, dd (9.0, 5.0)	Val-1,3,4,5, *N*MeTyr-1	Val-3,4,5, Ahp-OH
3	9.30, CH	0.27, m	Val-1,2,4,5	Val-2,4,5,NH, Ahp-OH
4	17.5, CH_3_	73.0, d (6.8)	Val-2,3,5	Val-2,3,NH, Ahp-OH, *N*MeTyr-*N*Me
5	19.5, CH_3_	0.85, d (6.8)	Val-2,3,4	Val-2,3, *N*MeTyr-*N*Me
NH	-	7.54, d (9.4)	*N*MeTyr-1	Val-2,3,4, *N*MeTyr-2,6,6′, Ahp-OH
*N*MeTyr	-	-	-	-
1	169.6, C	-	-	-
2	61.1, CH	9.41, m	*N*MeTyr-1,3,4,*N*Me, Leu-1	*N*MeTyr-3a,3b,5, 5′, Val-NH, Leu-2, Ahp-OH
3a	33.1,	2.68, d (13.9, 12.1)	*N*MeTyr-1,2,5,5′	*N*MeTyr-2,3a,5,5′
3b	CH_2_	3.10, brd (13.9)	*N*MeTyr-2,5,5′	*N*MeTyr-2,3b,5,5′
4	127.5, C	-	-	-
5,5′	130.2, CH	6.89, d (8.3)	*N*MeTyr-3,5′,5,6,6′,7	*N*MeTyr-2,3a,3b,6,6′ Leu-5
6,6′	115.5, CH	6.62, d (8.3)	*N*MeTyr-4,5,5′,6′,6,7	*N*MeTyr-5,5′,OH, Leu-5
7	156.2, C	-	-	-
OH	-	9.20 s	*N*MeTyr-5,5′,6,6′,7	*N*MeTyr-6,6′, Leu-5
NMe	30.6, CH_3_	2.70 s	*N*MeTyr-2, Leu-1	Val-4,5, Ahp-OH
Leu	-	-	-	-
1	171.0, C	-	-	-
2	47.9, CH	4.60, dd (10.5, 3.4)	Leu-1,3,4, Ahp-2,6	Leu-3a,4,5,6, *N*MeTyr-2
3a	38.7,	0.42, dt (10.1, 2.8)	Leu-2	Leu-2,3b, Ahp-6
3b	CH_2_	1.54, dt (10.8, 3.0)	Leu-2	Leu-3a, Ahp-6
4	23.8, CH	0.97, m	Leu-5,6	Leu-2, Ahp-3
5	22.3, CH_3_	0.49, d (6.4)	Leu-3,4,6	Leu-2, *N*MeTyr-3,5,5′,6,6′,OH
6	24.1, CH_3_	0.68, d (6.5)	Leu-2,3,4,5	Leu-2, Ahp-3
Ahp	-	-	-	-
2	169.3, C	-	-	-
3	49.2, CH	4.37, ddd (11.5, 9.2, 7.0)	Ahp-2,4, Gln-1	Ahp-5,NH, Leu-4,6
4a	22.0,	1.71, m	Ahp-2,5,6	Ahp-4b
4b	CH_2_	2.54, m	Ahp-3,5	Ahp-4a
5a	30.0,	1.71, m	Ahp-3	Ahp-3,6,OH
5b	CH_2_	1.71, m	Ahp-3	Ahp-3,6,OH
6	73.6, CH	4.89, brs	Ahp-3,4	Ahp-5,OH, Leu-3b
NH	-	7.34, d (9.2)	Ahp-3, Gln-1	Ahp-3, Gln-2
OH	-	6.02, d (2.8)	-	Ahp-5,6, *N*MeTyr -2, *N*Me, Val-2,3, 4,NH, Leu-2
Gln	-	-	-	-
1	170.2, C	-	-	-
2	52.3, CH	4.30, ddd (12.3, 8.5, 4.0)	Gln-1,3,4, Thr-1	Gln-3a,3b,4a,4b, NH, Ahp-NH
3a	27.0, CH_2_	1.66, m	Gln-1,2,4,5	Gln-2,3b,NH
3b	-	2.18, m	Gln-1,2,4	Gln-2,3a,NH
4a	31.9, CH_2_	2.02, m	Gln-2,3,5	Gln-2,4b,NH_2_(b)
4b		2.10, m	Gln-2,3,5	Gln-2,4a,NH_2_(b)
5	173.9, C	-	-	-
NH	-	8.50, d (8.5)	Gln-2,NH, Thr-1,2	Gln-2,3a,3b, Thr-2,3,4
NH_2_(a)	-	6.74, s	Gln-4,5	Gln-4a,4b
NH_2_(b)		7.20, s	Gln-5	
Thr	-	-	-	-
1	169.4, C	-	-	-
2	55.0, CH	4.67, brd (9.4)	Thr-1,3,4, Tyr-1	Thr-3,4, Gln-NH
3	72.1, CH	5.50, brq (6.5)	Thr-1,4 Val-1	Thr-2,4, Gln-NH
4	18.0, CH_3_	1.20, d (6.4)	Thr-1,2,3	Thr-2,3,NH
NH	-	8.25, d (9.4)	Tyr-1	Thr-4, Tyr-2,3a, 3b
Tyr	-	-	-	-
1	171.9, C	-	-	-
2	53.3, CH	4.71, m	Tyr-1,3,4, Hpla-1	Tyr-3a,3b,5,5′, NH, Thr-NH
3a	37.0, CH_2_	2.80, dd (13.9, 8.9)	Tyr-1,2,4,5,5′	Tyr-2,3b,5,5′,NH
3b		2.96, dd (13.9, 3.3)	Tyr-1,2,4,5,5′	Tyr-2,3a,5,5′,NH
4	127.4, C	-	-	-
5,5′	130.5, CH	6.98, d (8.3)	Tyr-3,5′,5,6,6′,7	Tyr-2,3a,3b,6,6′
6,6′	115.0, CH	6.60, d (8.3)	Tyr-4,5,5′,6,6′,7	Tyr-5,5′,OH
7	156.0, C	-	-	-
OH	-	9.11, s	Tyr-5,5′,6,6′,7	Tyr-6,6′
NH	-	7.70, d (8.2)	Tyr-1,2, Hpla-1	Tyr-2,3a,3b, Hpla-2,OH
Hpla	-	-	-	-
1	173.3, C	-	-	-
2	72.5, CH	3.94, ddd (9.0, 5.8, 2.4)	Hpla-1,3,4	Hpla-3a,3b,5,5′,2-OH
3a	39.7, CH_2_	2.45, dd (13.9, 9.0)	Hpla-2,4,5,5′	Hpla-2,3b
3b		2.78, dd (13.9, 2.4)	Hpla-2,4,5,5′	Hpla-2,3a
4	128.9, C	-	-	-
5,5′	130.4, CH	6.93, d (8.4)	Hpla-3,5′,5,6,6′,7	Hpla-2,6,6′
6,6′	114.9, CH	6.60, d (8.4)	Hpla-4,5,5′,6′,6,7	Hpla-5,5′,OH
7	155.7, C	-	-	-
2-OH	-	5.37, d (5.8)	Hpla-1,2,3	Hpla-2, Tyr-NH
7-OH	-	9.07, s	Hpla-5,5′,6,6′,7	Hpla-6,6′

^a^ 500 MHz for ^1^H, 125 MHz for ^13^C. ^b^ Type and assignment from an HSQC experiment. ^c^ HMBC correlations are from the carbon stated to the indicated proton(s). ^d^ Selected NOEs from ROESY experiment.

**Table 2:** NMR data of Aeruginosin TR642 (**2**) in DMSO-*d*_6_^a^.

Position	δ_C_, Type ^b^	δ_H_, mult.	LR CH Corr. ^c^	NOE Corr. ^d^
Hpla	-	-	-	-
1	172.92–173.40, C	-	-	-
2	72.28, 72.36, CH	4.06, m	Hpla-4,	Hpla-3a,3b,5, Ile-2,NH
3a	39.5, CH_2_	2.60–2.62, m	Hpla-1,2,4,5	Hpla-2,3b,5,2-OH, Ile-NH
3b		2.84–2.88, m	Hpla-1,2,4,5	Hpla-2,3a,5
4	128.21–128.47 C	-	-	-
5,5′	130.50–130.64, CH	6.98–7.00, d (8.6)	Hpla-2,3,5′,5	Hpla-2,3a,3b,6,6′
6,6′	114.86, 114.90, CH	6.61–6.63, d (8.6)	Hpla-4,5,5′,6′,6	Hpla-5,7-OH
7	155.88, C	-	-	-
2-OH	-	5.48–5.68, m	Hpla-1,2,3	-
7-OH	-	9.11, s	Hpla-6,6′,7	Hpla-6
Ile	-	-	-	-
1	169.25–169.74, C	-	-	-
2	53.12–53.60, CH	4.02–4.40, m	Hpla-1, Ile-1,3,4,6	Ile-3,4a,4b,5,6,NH, Choi-2, Hpla-2
3	34.96–37.42, CH	1.59–1.64, m	-	Ile-2,4a,4b,6,NH
4a	26.63–26.75, CH_2_	1.10–1.19, m	Ile-3,5,6	Ile-3,4b,5
4b		0.82–0.93, m	Ile-2,5,6	Ile-3,4a,5
5	11.68–12.14, CH_3_	0.71, m–0.81, t (6.8)	Ile-3,4	Ile-2,4a,4b, Hpla-5,6
6	13.52–14.29, CH_3_	0.66, brd (5.8)–0.73, d (6.4)	Ile-2,3,4	Ile-2,3,NH
NH	-	7.37–7.50, m	Hpla-1, Ile-2	Ile-2,3,6, Hpla-2,3a
Choi	-	-	-	-
1	171.3–172.22, C	-	-	-
2	58.42–59.33, CH	4.18, d (9.0)–4.71, m	Choi-1,3,3a,7a, Ile-1	Ile-2, Choi-3ax,3eq
3eq	30.65–33.61, CH_2_	2.21–2.27, m	Choi-1	Choi-2,3ax
3ax		1.34–1.68, m	Choi-1,7a	Choi-2,3eq
3a	34.60–31.99, CH	2.31–2.51, m	Choi-2,7a	-
4ax	21.78–22.84, CH_2_	1.44–2.03, m	Choi-5	Choi-6
4eq		2.15–2.49, m	Choi-7a	
5ax	26.01,	1.44, m	Choi-4,6	Choi-7ax
5eq	25.38, CH_2_	1.63–1.68, m	Choi-6	Choi-6,
6	70.65, CH	4.55, m, 4.49, dt (3.4,11.2)	Ac-1	Choi-4ax,5eq,7eq,7a
7ax	31.66,	0.97, m, 1.42, m	Choi-6,7a	Choi-5ax
7eq	34.56, CH_2_	2.48, m, 2.14, m	Choi-6	Choi-6, Ac-2
7a	56.45,	4.06, m	Choi-2	Choi-4ax,6
	56.55, CH	4.34, m		
Ac 1	169.83, C	-	Choi-6	-
2	21.21,	1.99, s	Ac-1	Choi-7eq
	21.16, CH_3_	1.94, s		
Ddha	-	-	-	-
1	74.52–75.76, CH	5.09–5.37, m	Ddha-5	Ddha-1-OH,2,2-NH,3a,3b,7-NH_2_
2	45.28–46.83, CH	3.76–3.97, m	Ddha-3	Ddha-1,4,3b
3a	21.69–22.70, CH_2_	2.15–2.54, m	Ddha-2,4,5	Ddha-1,4
3b		1.88–2.02, m	Ddha-1,2,4,5	Ddha-1,2,4
4	107.38–108.86, CH	5.21, 5.32, 5.16, 5.23, 5.13, 5.17, 5.21, 5.21 m	Ddha-2	Ddha-2,3a,3b,5
5	121.45–122.19, CH	6.36, d (6.7)–6.50, m	Ddha-1,3,4,7	Ddha-4,7-NH_2_
1-OH	-	6.36–6.88, brs	-	Ddha-7-NH_2_
2-NH	-	7.31, m–8.39, d (7.1)	Choi-1	Ddha-1
7	154.70–155.68, C	-	-	-
7-NH_2_	-	7.90, brs	-	Ddha-1,1-OH,5

**^a^** 500 MHz for ^1^H, 125 MHz for ^13^C. ^b^ Type and assignment from an HSQC experiment. ^c^ HMBC correlations are from the proton(s) stated to the indicated carbon. ^d^ Selected NOEs from ROESY experiment.

## Supplementary Materials

The following are available online at www.mdpi.com/1660-3397/15/12/371/s1. Figure S1 presenting the structures and Tables S1 and S2 containing the 1H and ^13^C NMR data, respectively, of the isolation artifact micropeptins derivatives. 1D (^1^H, ^13^C) and 2D NMR (HSQC, HMBC, COSY, TOCSY, ROESY) spectra and HR MS data of compounds **1** and **2**.
