# Supplementary material for: Inhibitors of Serine Proteases from a Microcystis sp. Bloom Material Collected from Timurim Reservoir, Israel
Source: Mar Drugs. 2017 Dec 1;15(12):371. doi: 10.3390/md15120371 (PMC5742831; doi:10.3390/md15120371)
Supplement: Supplementary file 1 [file marinedrugs-15-00371-s001.pdf]

Inhibitors of Serine Proteases from a *Microcystis* sp. Bloom-Material collected from Timurim Reservoir, Israel

*Rawan Hasan-Amer and Shmuel Carmeli**

Raymond and Beverly Sackler School of Chemistry, Raymond and Beverly Sackler Faculty of Exact Sciences,

Tel-Aviv University, Ramat Aviv, Tel-Aviv 69978, Israel.

Supplementary Material

Table of content

S1. Figure S1. Structures of the Metabolites and Isolation Artifacts Isolated from the Extract of IL-428
S2. Table S1. ^{1}H NMR Data *of **1** and its isolation artifacts **3-8** in DMSO-d_6*
S3. Table S2. ^{13}C NMR Data of **1** and its isolation artifacts **3-8** in DMSO-d_6
S4. ^{1}H NMR Spectrum of Micropeptin TR1058 (**1**) in DMSO-d_6
S5. ^{13}C NMR Spectrum of Micropeptin TR1058 (**1**) in DMSO-d_6
S6. HSQC Spectrum Micropeptin TR1058 (**1**) in DMSO-d_6
S7. HMBC Spectrum of Micropeptin TR1058 (**1**) in DMSO-d_6
S8. COSY Spectrum of Micropeptin TR1058 (**1**) in DMSO-d_6
S9. TOCSY Spectrum of Micropeptin TR1058 (**1**) in DMSO-d_6
S10. ROESY Spectrum of Micropeptin TR1058 (**1**) in DMSO-d_6
S11. HR ESI MS data of Micropeptin TR1058 (**1**)
S12. ^{1}H NMR Spectrum of Aeruginosin TR642 (**2**) in DMSO-d_6
S13. ^{13}C NMR Spectrum of Aeruginosin TR642 (**2**) in DMSO-d_6
S14. HSQC Spectrum of Aeruginosin TR642 (**2**) in DMSO-d_6
S15. HMBC Spectrum of Aeruginosin TR642 (**2**) in DMSO-d_6
S16. COSY Spectrum of Aeruginosin TR642 (**2**) in DMSO-d_6
S17. TOCSY Spectrum of Aeruginosin TR642 (**2**) in DMSO-d_6
S18. ROESY Spectrum of Aeruginosin TR642 (**2**) in DMSO-d_6
S19. HR ESI MS data of Aeruginosin TR642 (**2**)

S1. Figure S1. Structures of the Metabolites and Isolation Artifacts Isolated from the Extract of IL-428

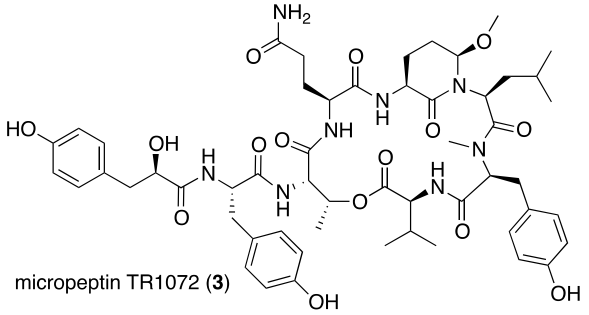

micropeptin TR1072 (**3**)

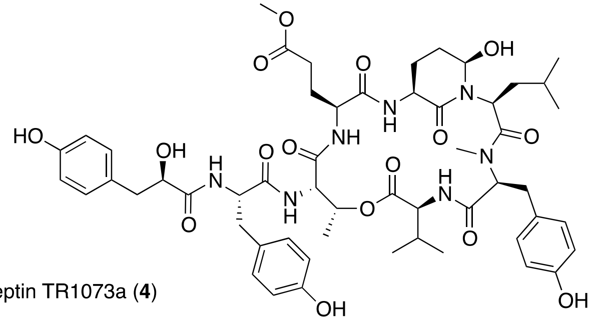

micropeptin TR1073a (**4**)

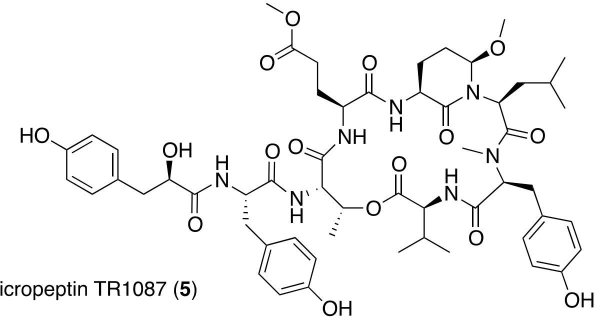

micropeptin TR1087 (**5**)

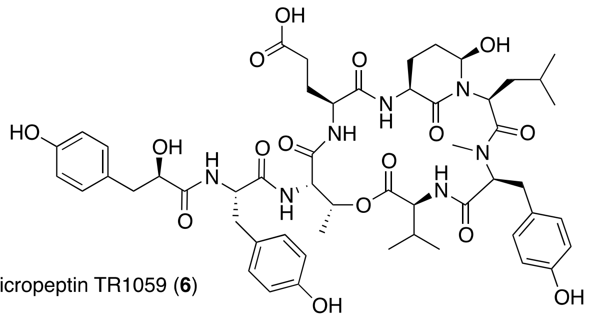

micropeptin TR1059 (**6**)

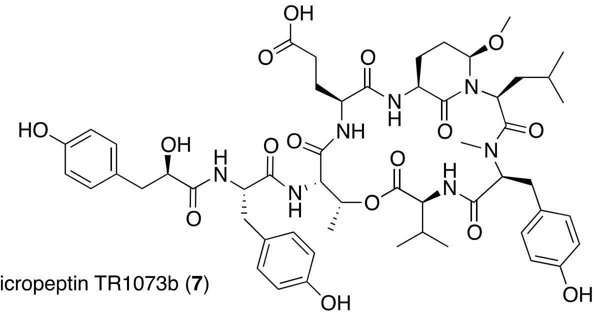

micropeptin TR1073b (**7**)

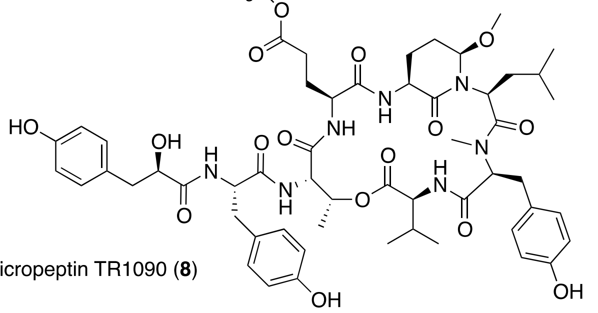

micropeptin TR1090 (**8**)

S2. Table S1. ^{1}H NMR Data of **1** and its isolation artifacts **3-8** in DMSO-d_6

Position		**1**	**3**	**4**	**5**	**6**	**7**	**8**
Val	2	4.73 m	4.75 m	4.75 m	4.77 m	4.73 m	4.77 m	4.77 m
	3	2.06 m	2.07 m	2.06 m	2.07 m	2.05 m	2.07 m	2.07 m
	4	0.73 d	0.79 d	0.71 d	0.78 d	0.72 d	0.78 d	0.78 d
	5	0.85 d	0.89 d	0.85 d	0.89 d	0.85 d	0.89 d	0.89 d
	NH	7.53 d	7.53 d	7.49 d	6.78 d	7.51 d	6.75 d	6.75 d
N-MeTyr	2	4.91 m	4.97 m	4.91 m	4.96 m	4.89 m	4.96 m	4.96 m
	3a	2.66 d	2.66 d	2.68 d	2.67 d	2.64 d	2.67 d	2.67 d
	3b	3.09 d	3.10 d	3.10 d	3.10 d	3.10 d	3.10 d	3.10 d
	5,5'	6.88 d	6.89 d	6.89 d	6.89 d	6.89 d	6.89 d	6.89 d
	6,6'	6.62 d	6.64 d	6.64 d	6.64 d	6.64 d	6.62 d	6.62 d
	OH	9.20 s	9.14 s	9.12 s	9.15 brs	9.21 s	9.15 brs	9.15 brs
	*N*Me	2.70 s	2.72 s	2.70 s	2.71 s	2.70 s	2.71 s	2.71 s
Leu	2	4.59 dd 10.0,4.5	4.64 dd	4.60 dd	4.64 dd	4.58 dd	4.64 m	4.64 m
	3a	0.41 dt 10.0,1.5	0.31 dt	0.40 dt	0.29 dt	0.40 dt	0.29 dt	0.29 dt
	3b	1.54 dt 10.0,1.5	1.62 dt	1.54 dt	1.62 dt	1.54 dt	1.62 m	1.62 m
	4	0.96 m	0.99 m	0.96 m	0.99 m	0.96 m	0.99 m	0.99 m
	5	0.49 d	0.49 d	0.49 d	0.48 d	0.48 d	0.48 d	0.48 d
	6	0.68 d	0.70 d	0.68 d	0.69 d	0.69 d	0.69 d	0.69 d
Ahp/Amp	3	4.37 ddd	4.41	4.39	4.43	4.38	4.43 m	4.43 m
	4a	1.71 m	1.72 m	1.71 m	1.72 m	1.71 m	1.72 m	1.72 m
	4b	2.54 m	2.35 m	2.30 m	2.35 m	2.48 m	2.35 m	2.35 m
	5a	1.71 m	1.60 m	1.71 m	1.59 m	1.70 m	1.59 m	1.59 m
	5b	1.71 m	2.04 m	1.71 m	2.03 m	1.70 m	2.03 m	2.03 m
	6	4.88 brs	4.41 s	4.88 s	4.40 s	4.88 s	4.40 s	4.40 s
	NH	7.34 d	7.34 d	7.35 d	7.20 d	7.35 d	7.20 d	7.20 d
	*O*Me	----	3.02 s	----	3.01 s	----	3.01 s	3.01 s
	OH	6.02 d	----	6.02 d	----	6.02 d	----	----
Gln/Glu/ *O*Me Glu	2	4.29 ddd	4.31 m	4.35 m	4.35 m	4.34 m	4.35 m	4.35 m
	3a	1.66 m	1.65 m	1.65 m	1.65 m	1.61 m	1.65 m	1.65 m
	3b	2.18 m	2.19 m	2.30 m	2.30 m	2.22 m	2.30 m	2.30 m
	4a	2.02 m	2.11 m	2.32 m	2.30 m	2.21 m	2.30 m	2.30 m
	4b	2.10 m	2.02 m	2.30 m	2.30 m	2.18 m	2.30 m	2.30 m
	NH	8.49 d	8.55 d	8.52 d	8.57 d	8.49 d	8.53 d	8.53 d

	*O*Me	----	----	3.56 s	3.56 s	----	----	----
	NH_2(a)	6.74 s	7.21 d	----	----	----	----	----
	NH_2(b)	7.19 s	6.75 s	----	----	----	----	----
Thr	2	4.67 d	4.70 d	4.67 d	4.68 d	4.67 d	4.68 d	4.68 d
	3	5.49 q	5.53 q	5.50 q	5.52 q	5.49 q	5.52 q	5.52 q
	4	1.20 d	1.21 d	1.19 d	1.19 d	1.19 d	1.19 d	1.19 d
	NH	8.25 d	8.29 d	8.31 d	8.35 d	8.28 d	8.34 d	8.34 d
Tyr	2	4.71 m	4.72 m	4.73 m	4.74 m	4.70 m	4.74 m	4.74 m
	3a	2.82 m	2.81 m	2.81 m	2.77 m	2.77 m	2.77 m	2.77 m
	3b	2.96 m	2.96 m	2.95 m	2.95 m	2.95 m	2.95 m	2.95 m
	5,5'	6.92 d	6.93 d	6.92 d	6.93 d	6.92 d	6.89 d	6.89 d
	6,6'	6.61 d	6.62 d	6.62 d	6.62 d	6.62 d	6.64 d	6.64 d
	OH	9.07 s	9.14 s	9.12 brs	9.15 brs	9.08 s	9.15 brs	9.15 brs
	NH	7.69 d	7.71 d	7.69 d	7.69 d	7.67 d	7.67 d	7.67 d
Hpla	2	3.94 ddd	3.96	3.94	3.96	3.95	3.96	3.96
	3a	2.44 m	2.44 m	2.45 m	2.45 m	2.43 m	2.45 m	2.45 m
	3b	2.79 m	2.81 m	2.80 m	2.80 d	2.77 d	2.80 d	2.80 d
	5,5'	6.97 d	6.98 d	6.96 d	6.95 d	6.95 d	6.92 d	6.92 d
	6,6'	6.60 d	6.60 d	6.60 d	6.60 d	6.60 d	6.60 d	6.60 d
	2-OH	5.36 d	5.36 d	5.38 d	5.36 d	5.37 d	5.35 d	5.35 d
	7-OH	9.11 s	9.16 brs	9.12 brs	9.15 brs	9.11 s	9.15 brs	9.15 brs

S3. Table S2. ^{13}C NMR Data of **1** and its isolation artifacts **3-8** in DMSO-d_6

Position		**1** δ$_C$	**3** δ$_C$	**4** δ$_C$	**5** δ$_C$	**6** δ$_C$	**7** δ$_C$	**8** δ$_C$
Val	1	172.5 s	172.3 s	172.5 s	172.4 s	172.5 s	172.4 s	172.4 s
	2	56.2 d	56.0 d	56.1 d	56.0 d	56.2 d	56.0 d	56.0 d
	3	30.9 d	31.3 d	30.8 d	31.4 d	30.9 d	31.4 d	31.4 d
	4	17.8 q	17.8 q	17.9 q	17.8 q	18.0 q	17.8 q	17.8 q
	5	19.5 q	19.4 q	19.5 q	19.5 q	19.5 q	19.4 q	19.4 q
N-MeTyr	1	169.3 s	169.3 s	169.4 s	169.5 s	169.4 s	169.5 s	169.5 s
	2	61.1 d	61.3 d	61.1 d	61.4 d	61.1 d	61.3 d	61.3 d
	3	33.1 t	33.0 t	33.1 t	33.0 t	33.1 t	33.0 t	33.0 t
	4	127.5 s	127.4 s	127.4 s	127.4 s	127.5 s	127.4 s	127.4 s
	5,5'	130.5 d	130.2 d	130.5 d	130.6 d	130.0 d	130.5 d	130.5 d
	6,6'	115.5 d	115.0 d	115.0 d	115.1 d	115.1 d	115.0 d	115.0 d
	7	156.2 s	156.2 s	156.0 s	156.0 s	156.0 s	156.0 s	156.0 s
	N	30.6 q	30.5 q	30.6 q	30.7 q	30.6 q	30.6 q	30.6 q
Leu	1	171.0 s	170.9 s	171.0 s	171.0 s	171.0 s	171.0 s	171.0 s
	2	47.9 d	47.4 d	47.9 d	47.5 d	47.9 d	47.5 d	47.5 d
	3	38.7 t	38.0 t	38.7 t	38.1 t	38.7 t	38.0 t	38.0 t
	4	23.8 d	23.8 d	23.8 d	23.8 d	23.8 d	23.8 d	23.8 d
	5	22.3 q	22.1 q	22.3 q	22.2 q	22.3 q	22.2 q	22.2 q
	6	24.1 q	24.0 q	24.0 q	24.0 q	24.1 q	24.0 q	24.0 q
Ahp/Amp	2	169.7 s	169.1 s	169.6 s	169.1 s	169.7 s	169.1 s	169.1 s
	3	49.2 d	49.1 d	49.1 d	49.4 d	49.2 d	49.3 d	49.3 d
	4	22.0 t	22.0 t	22.0 t	22.0 t	22.0 t	22.0 t	22.0 t
	5	30.0 t	23.8 t	30.0 t	23.7 t	30.0 t	23.8 t	23.8 t
	6	73.6 d	82.6 d	73.6 d	82.6 d	73.7 d	82.6 d	82.6 d
	O	----	55.5 q	----	55.6 q	----	55.5 q	55.5 q
Gln/Glu/	1	170.2 s	170.3 s	170.0 s	170.2 s	170.2 s	170.1 s	170.2 s
*O*Me Glu	2	52.3 d	52.1 d	51.6 d	51.6 d	51.9 d	51.7 d	51.6 d

	3	27.0 t	27.0 t	26.1 t	26.1 t	26.3 t	26.3 t	26.3 t
	4	31.9 t	31.8 t	30.0 t	30.0 t	30.3 t	30.0 t	30.0 t
	CO	173.9 s	173.9 s	173.0 s	173.0 s	174.2 s	174.1 s	173.0 s
	O	----	----	51.6 q	51.6 q	----	----	----
Thr	1	169.3 s	169.5 s	169.3 s	169.4 s	169.4 s	169.4 s	169.4 s
	2	55.0 d	55.0 d	55.0 d	55.0 d	55.0 d	55.0 d	55.0 d
	3	72.1 d	72.1 d	72.1 d	72.1 d	72.2 d	72.1 d	72.1 d
	4	17.5 q	17.4 q	17.4 q	17.4 q	17.5 q	17.4 q	17.4 q
Tyr	1	171.9 s	172.0 s	171.8 s	171.9 s	171.9 s	171.9 s	171.9
	2	53.3 d	53.3 d	53.2 d	53.2 d	53.2 d	53.2 d	53.2 d
	3	37.0 t	37.0 t	37.1 t	37.1 t	37.1 t	37.1 t	37.1 t
	4	127.4 s	127.3 s	127.4 s	127.4 s	127.4 s	127.4 s	127.4 s
	5,5'	130.4 d	130.5 d	130.2 d	130.3 d	130.3 d	130.2 d	130.2 d
	6,6'	115.0 d	115.6 d	115.5 d	115.7 d	115.5 d	115.6 d	115.6 d
	7	155.7 s	155.7 s	156.2 s	156.3 s	156.3 s	156.3 s	156.3 s
Hpla	1	173.3 s	173.3 s	173.2 s	173.3 s	173.3 s	173.3 s	173.3 s
	2	72.5 d	72.5 d	72.5 d	72.6 d	72.6 d	72.5 d	72.5 d
	3	39.8 t	39.2 t	39.1 t	39.2 t	39.2 t	39.2 t	39.2 t
	4	128.9 s	128.8 s	128.8 s	128.9 s	128.9 s	128.9 s	128.9 s
	5,5'	130.2 d	130.4 d	130.4 d	130.5 d	130.5 d	130.4 d	130.4 d
	6,6'	114.8 d	114.9 d	114.9 d	114.9 d	114.9 d	114.9 d	114.9 d
	7	155.9 s	156.0 s	155.7 s	155.8 s	155.8 s	155.7 s	155.7 s

S4. ^{1}H NMR Spectrum of Micropeptin TR1058 (**1**) in DMSO-d_6

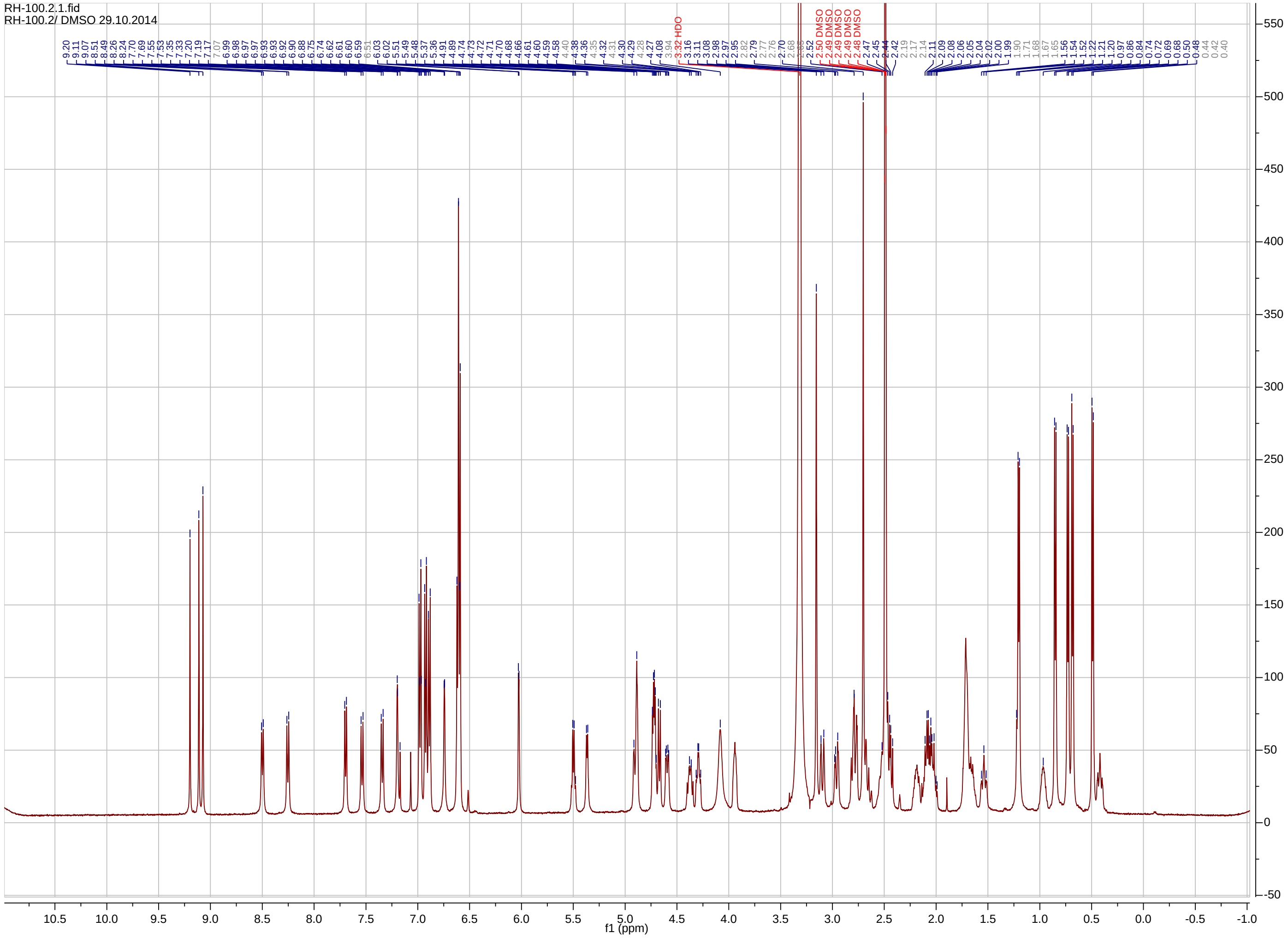

S5. ^{13}C NMR Spectrum of Micropeptin TR1058 (**1**) in DMSO-d_6

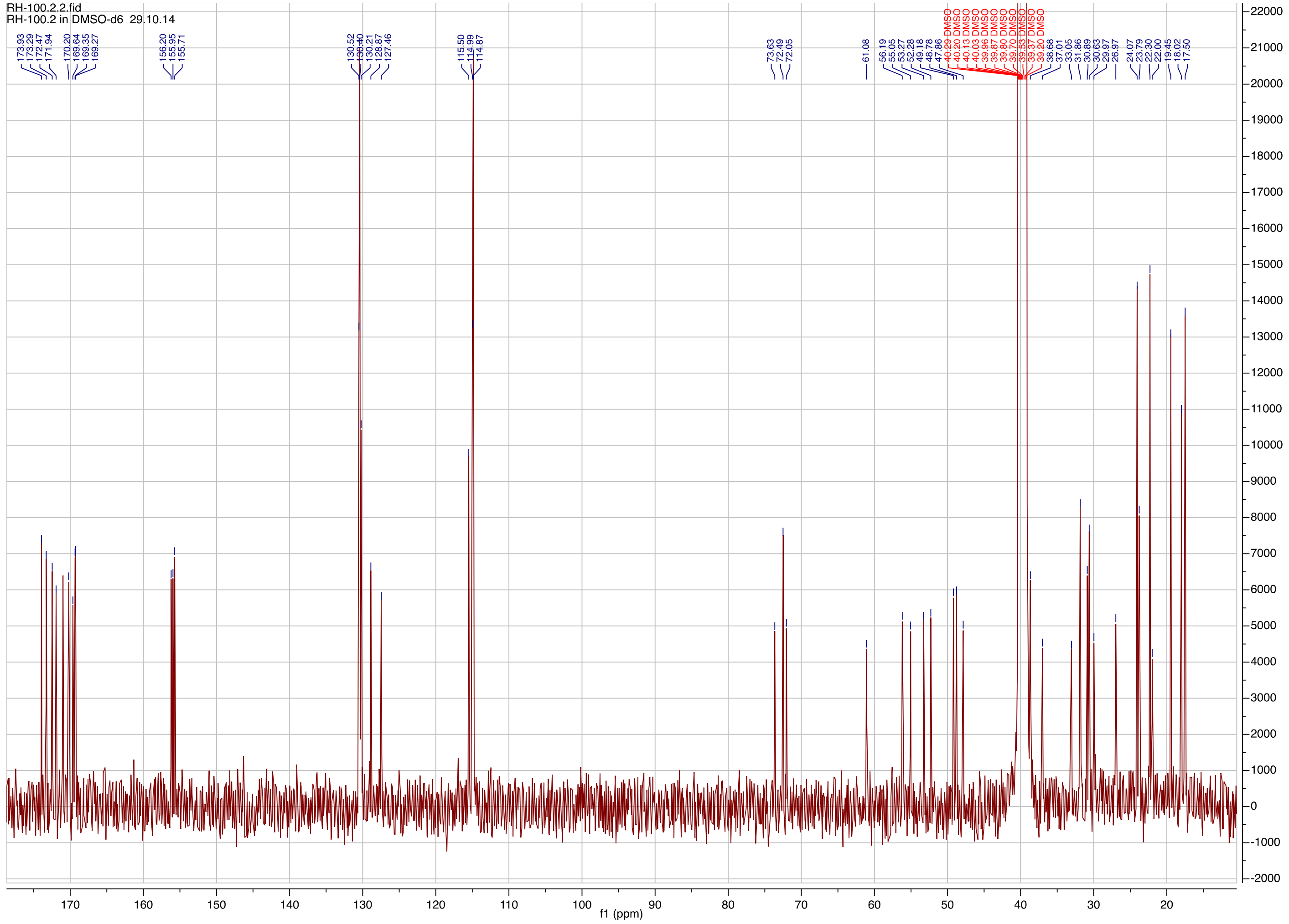

S6. HSQC Spectrum Micropeptin TR1058 (**1**) in DMSO-d_6 (CH, CH_3, CH_2)

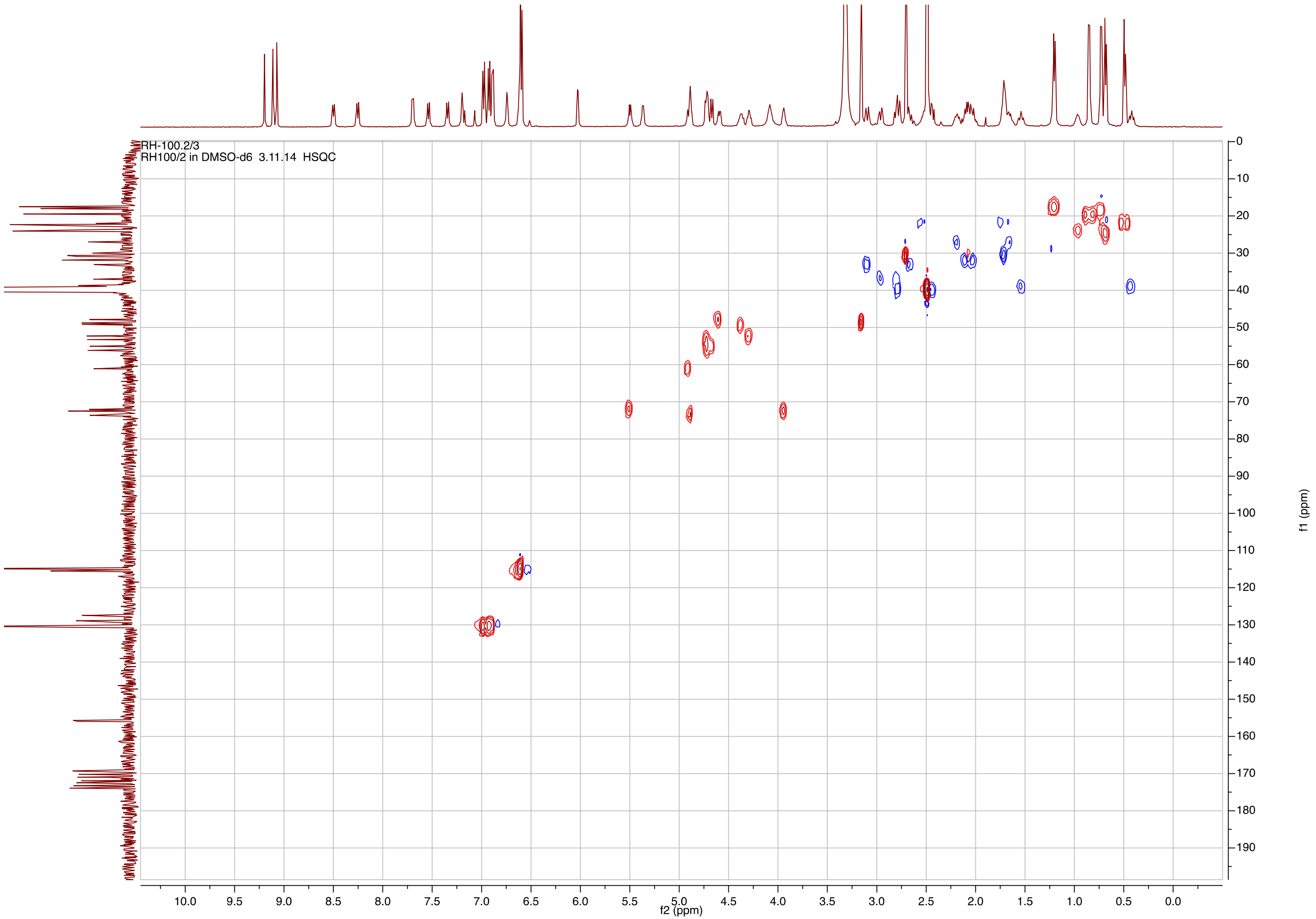

S7. HMBC Spectrum of Micropeptin TR1058 (**1**) in DMSO-d_6

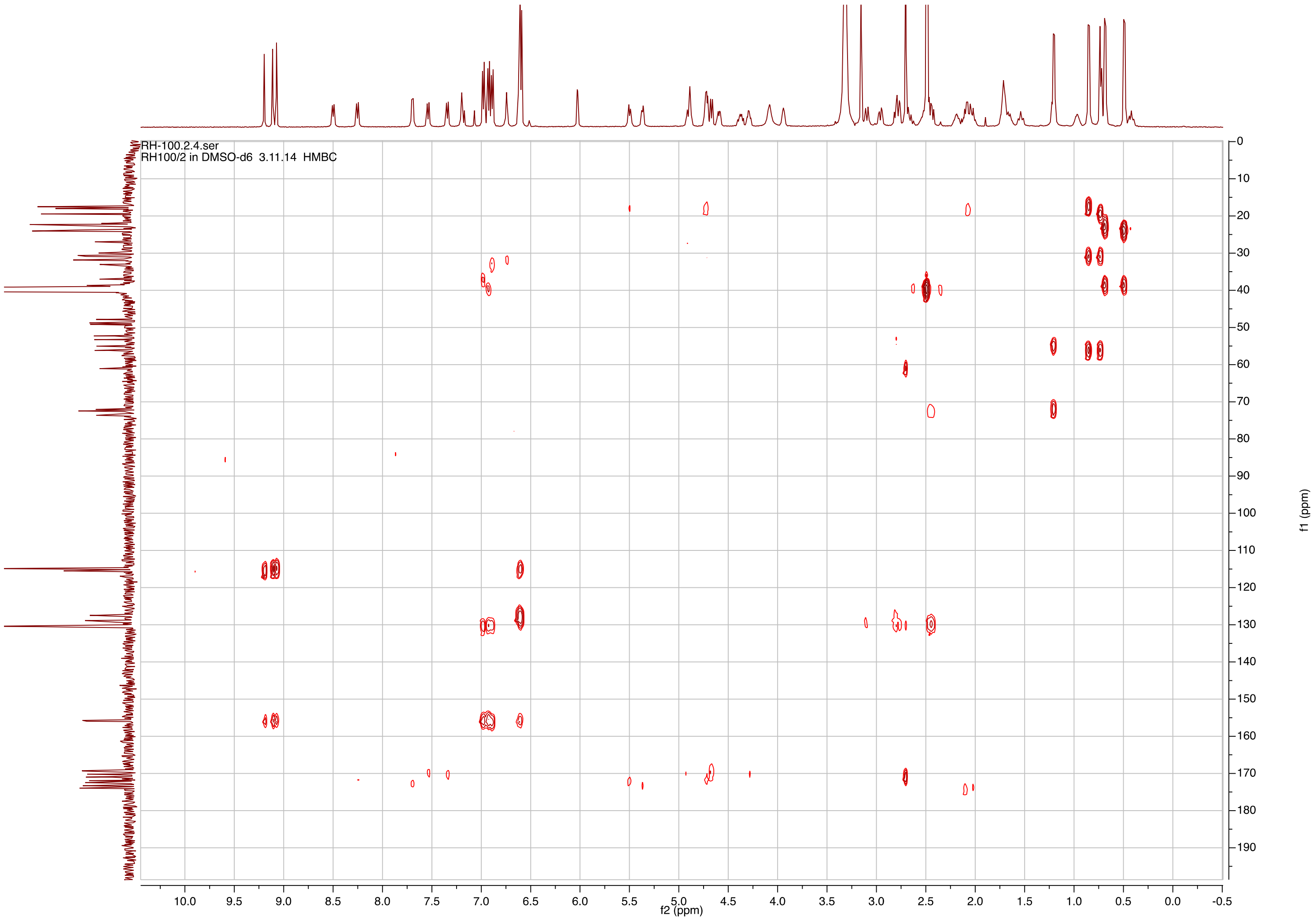

S8. COSY Spectrum of Micropeptin TR1058 (**1**) in DMSO-d_6

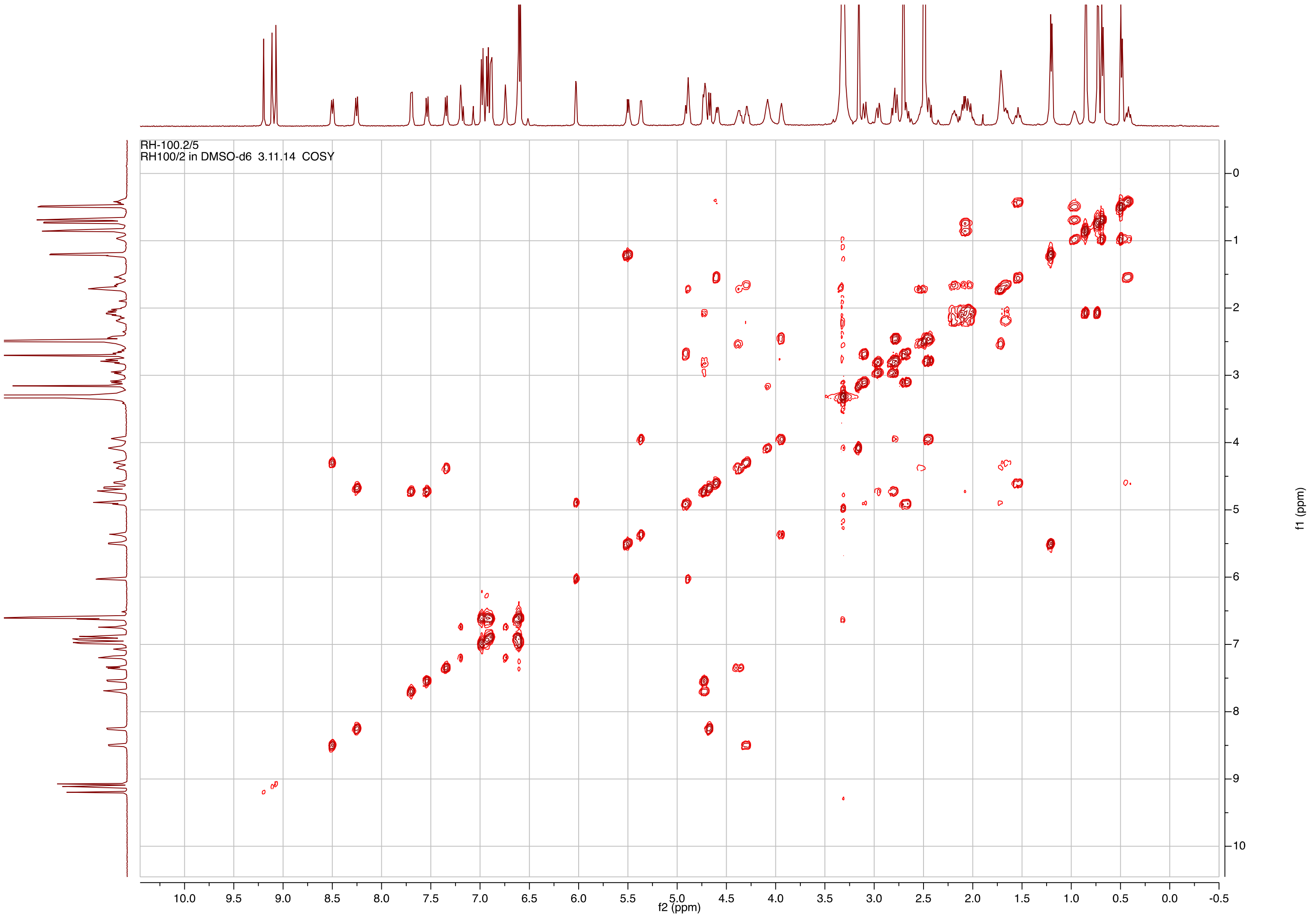

S9. TOCSY Spectrum of Micropeptin TR1058 (**1**) in DMSO-d_6

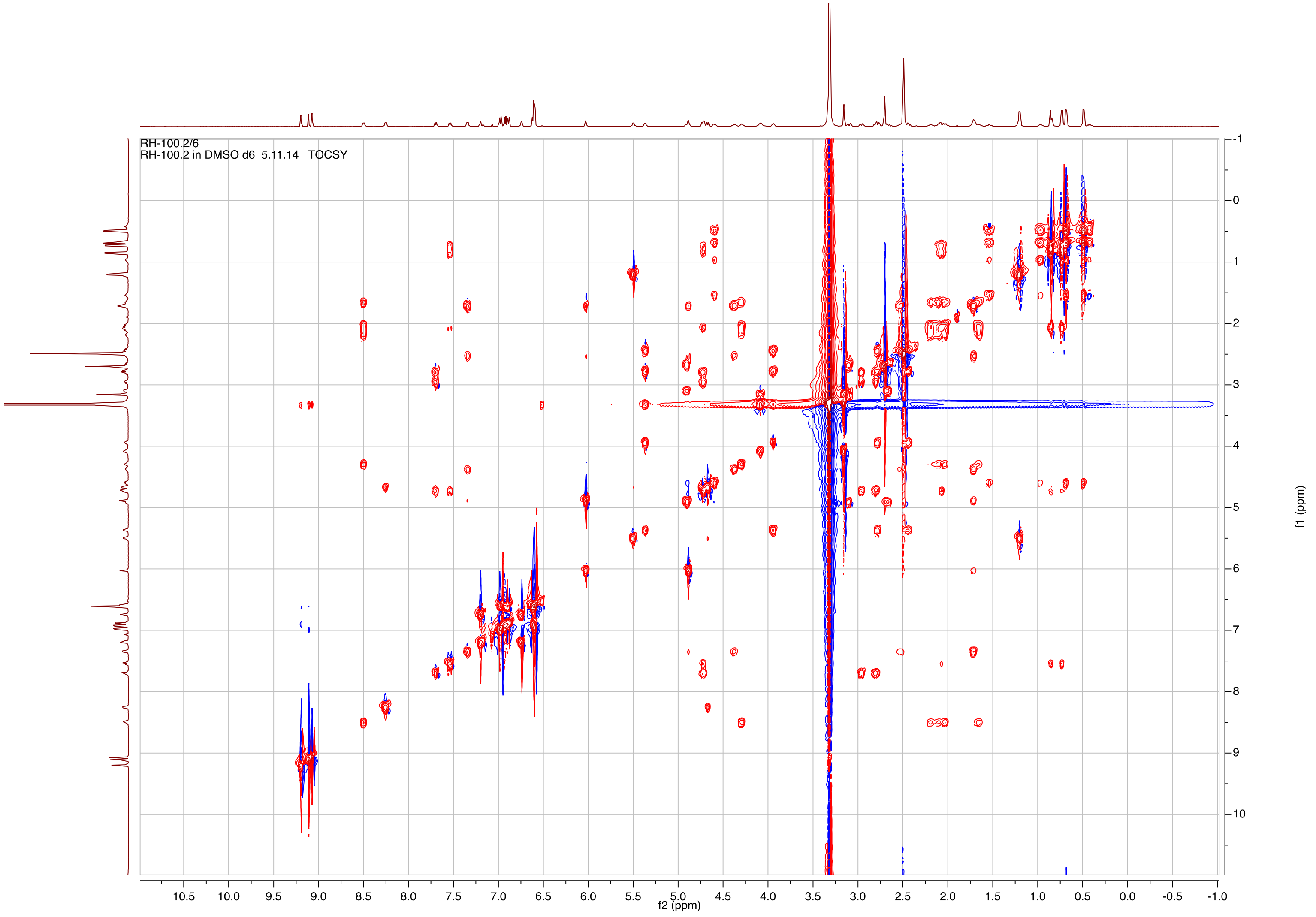

S10. ROESY Spectrum of Micropeptin TR1058 (**1**) in DMSO-d_6 (NOE correlation)

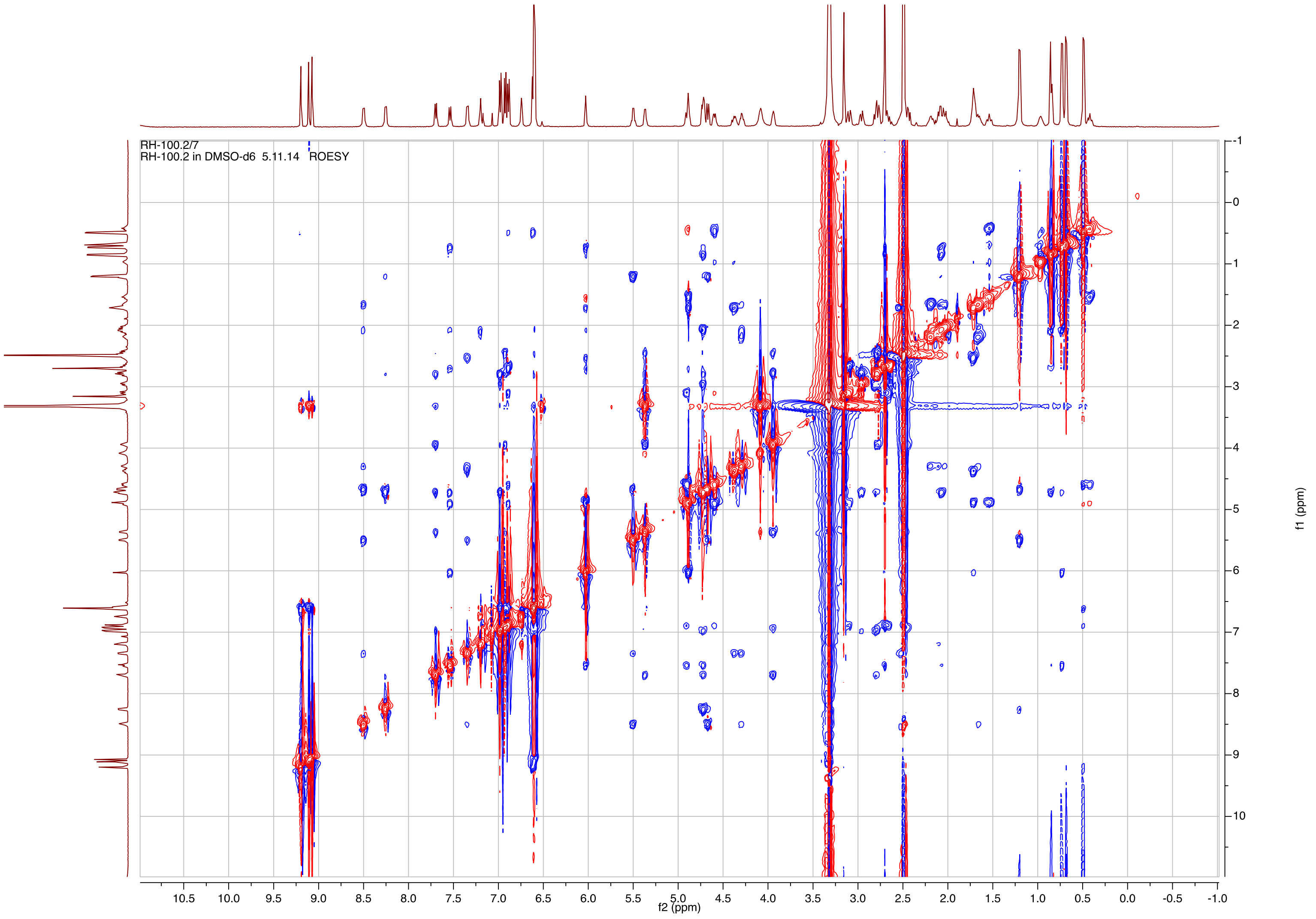

S11. HR ESI MS data of Micropeptin TR1058 (**1**)

Elemental Composition Report **Page 1**

Single Mass Analysis
Tolerance = 5.0 PPM / DBE: min = -1.5, max = 50.0
Element prediction: Off
Number of isotope peaks used for i-FIT = 3

Monoisotopic Mass, Even Electron Ions
234 formula(e) evaluated with 6 results within limits (up to 5 closest results for each mass)
Elements Used:
C: 50-60 H: 65-80 N: 0-15 O: 0-20

RH-100.2 Rawan Hassan
carmeli960a 85 (3.241) Cm (85:87) 1: TOF MS ES- 2.28e+002

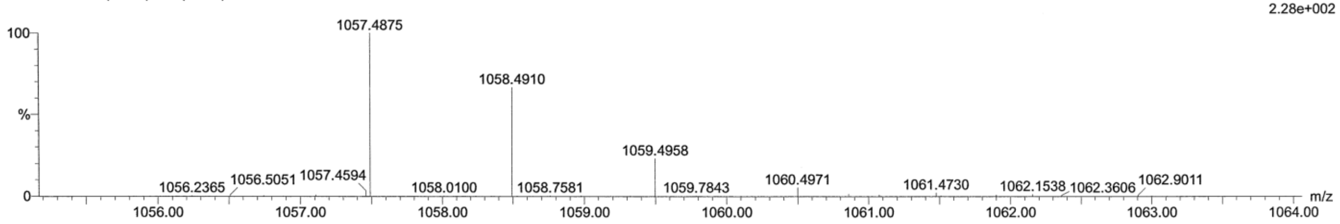

Mass	Calc. Mass	mDa	PPM	DBE	i-FIT	i-FIT (Norm)	Formula
Minimum:				-1.5			
Maximum:		5.0	5.0	50.0			
1057.4875	1057.4869	0.6	0.6	18.5	17.3	1.5	C52 H73 N4 O19
	1057.4882	-0.7	-0.7	23.5	17.1	1.4	C53 H69 N8 O15
	1057.4896	-2.1	-2.0	28.5	17.2	1.4	C54 H65 N12 O11
	1057.4909	-3.4	-3.2	22.5	17.6	1.8	C57 H73 N2 O17
	1057.4923	-4.8	-4.5	27.5	18.0	2.2	C58 H69 N6 O13

S12. ^{1}H NMR Spectrum of Aeruginosin TR642 (**2**) in DMSO-d_6

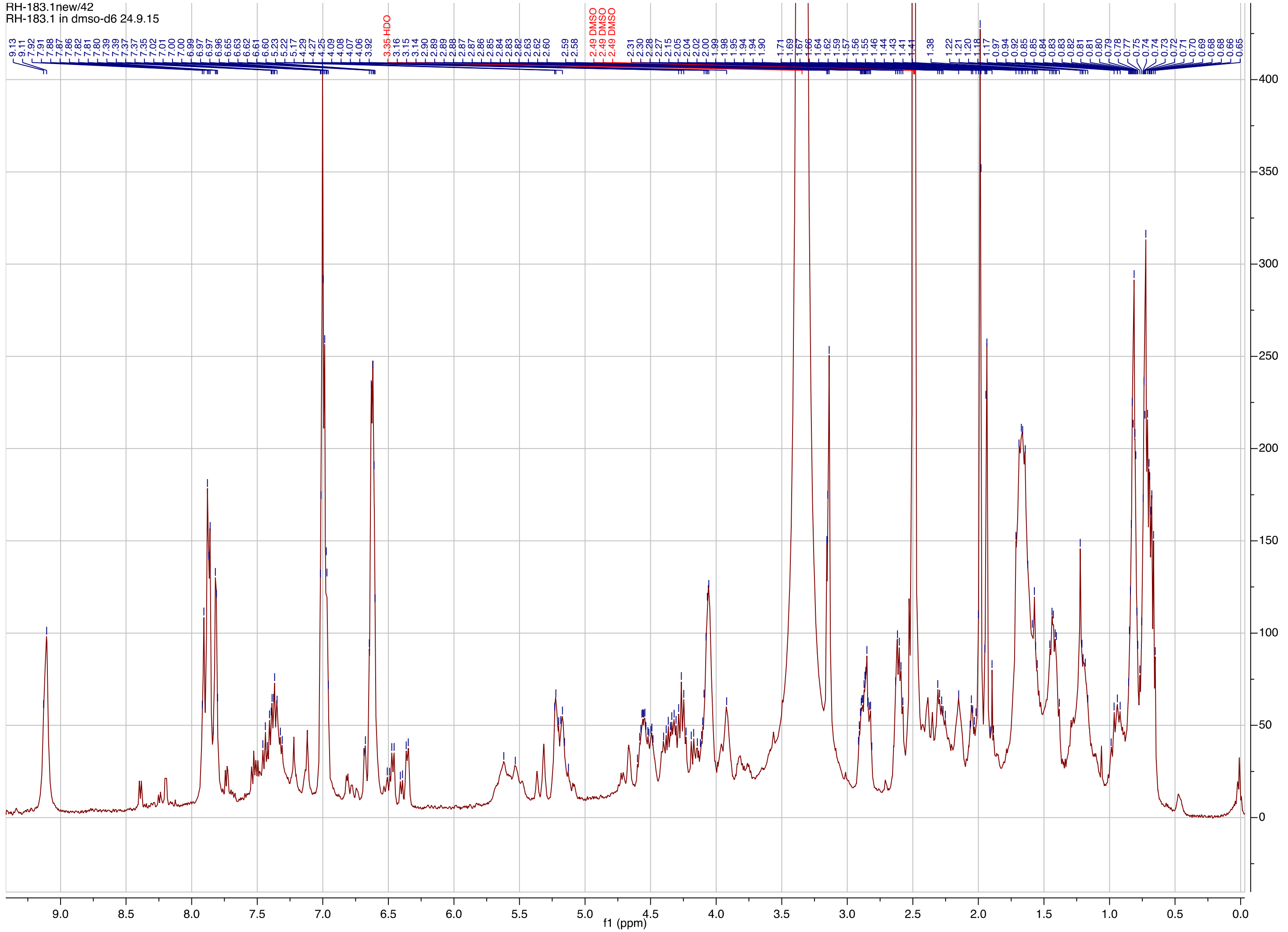

S13. ^{13}C NMR Spectrum of Aeruginosin TR642 (**2**) in DMSO-d_6

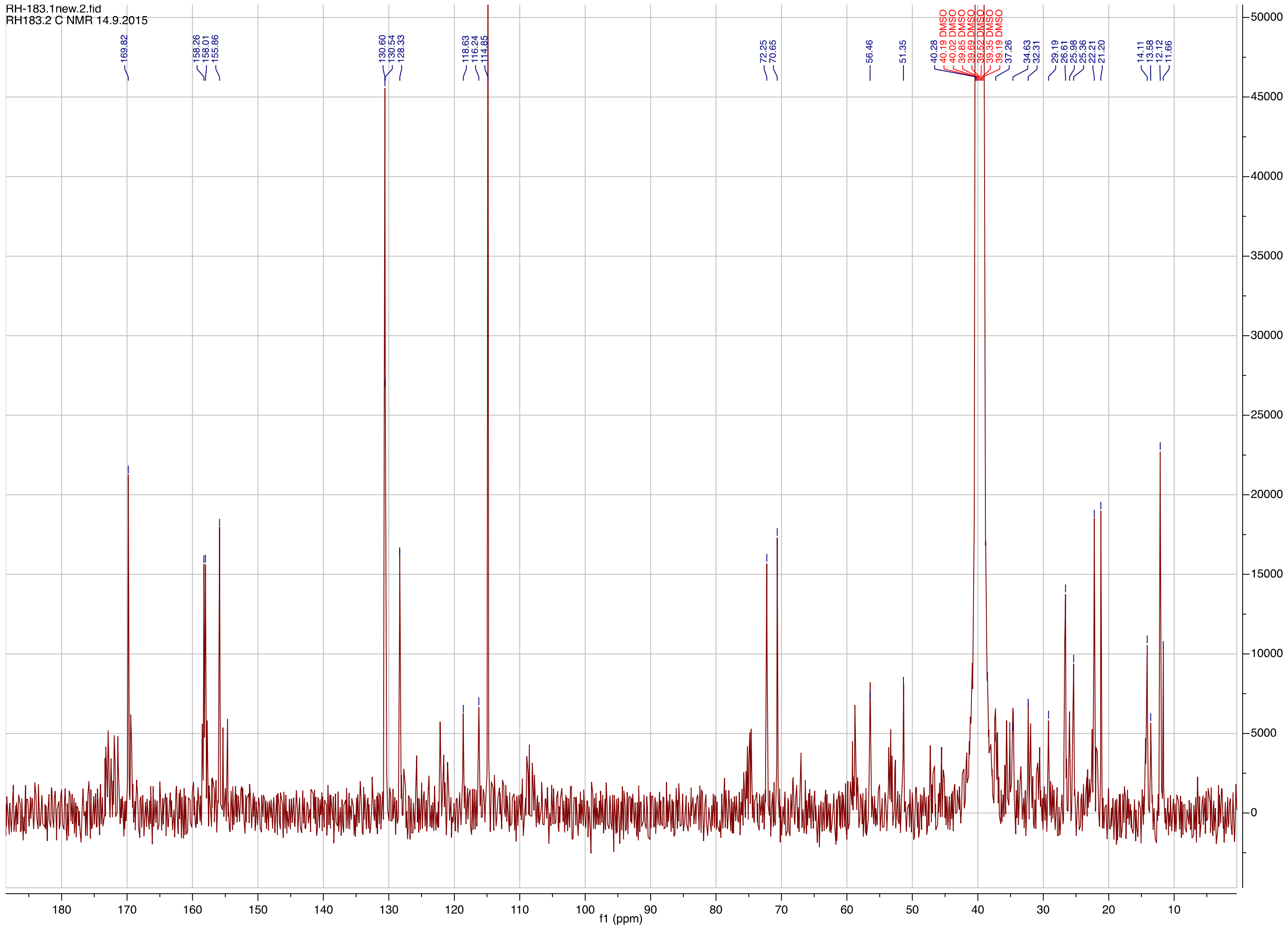

S14. HSQC Spectrum of Aeruginosin TR642 (**2**) in DMSO-d_6 (CH, CH_3, CH_2)

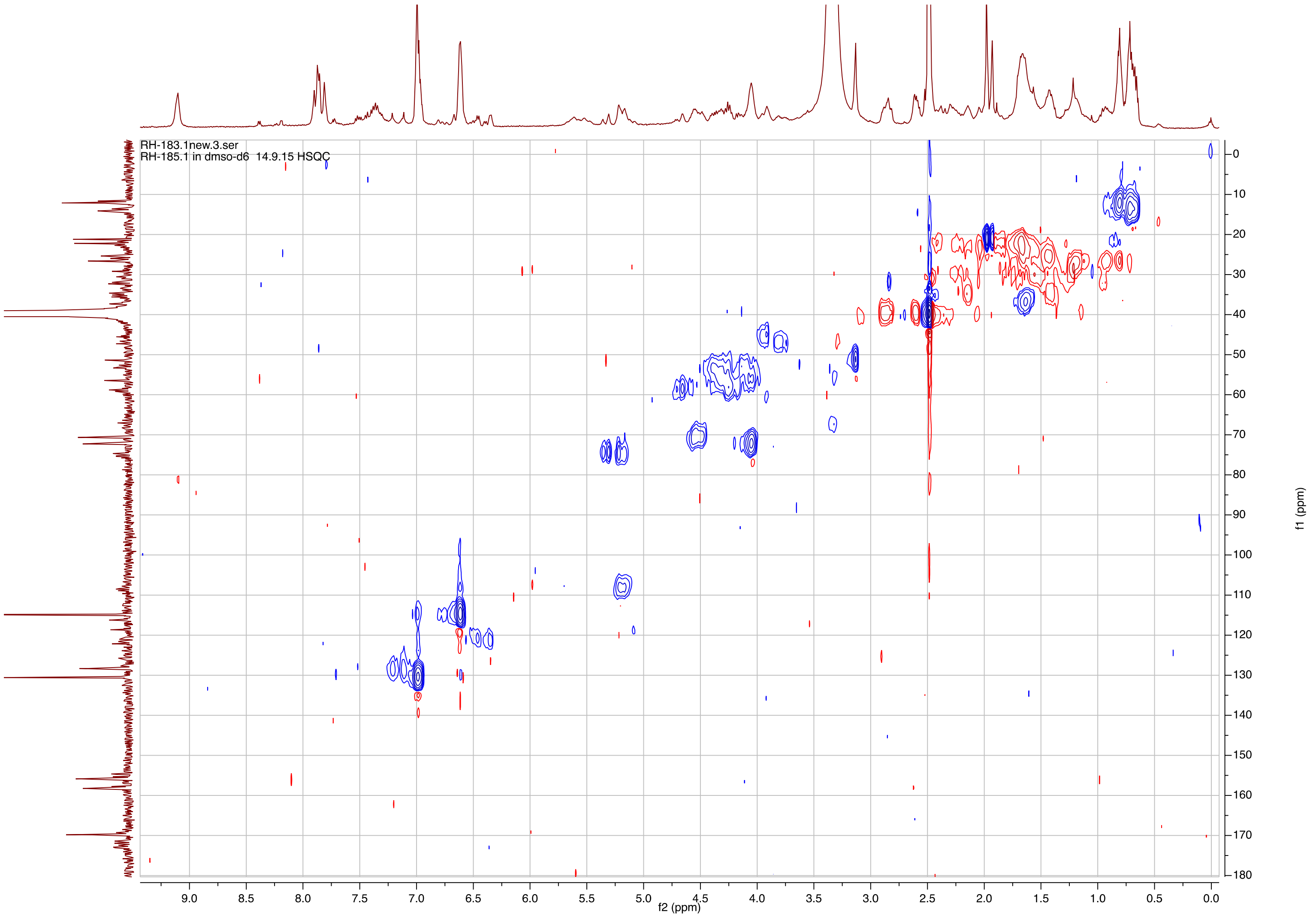

S15. HMBC Spectrum of Aeruginosin TR642 (**2**) in DMSO-d_6

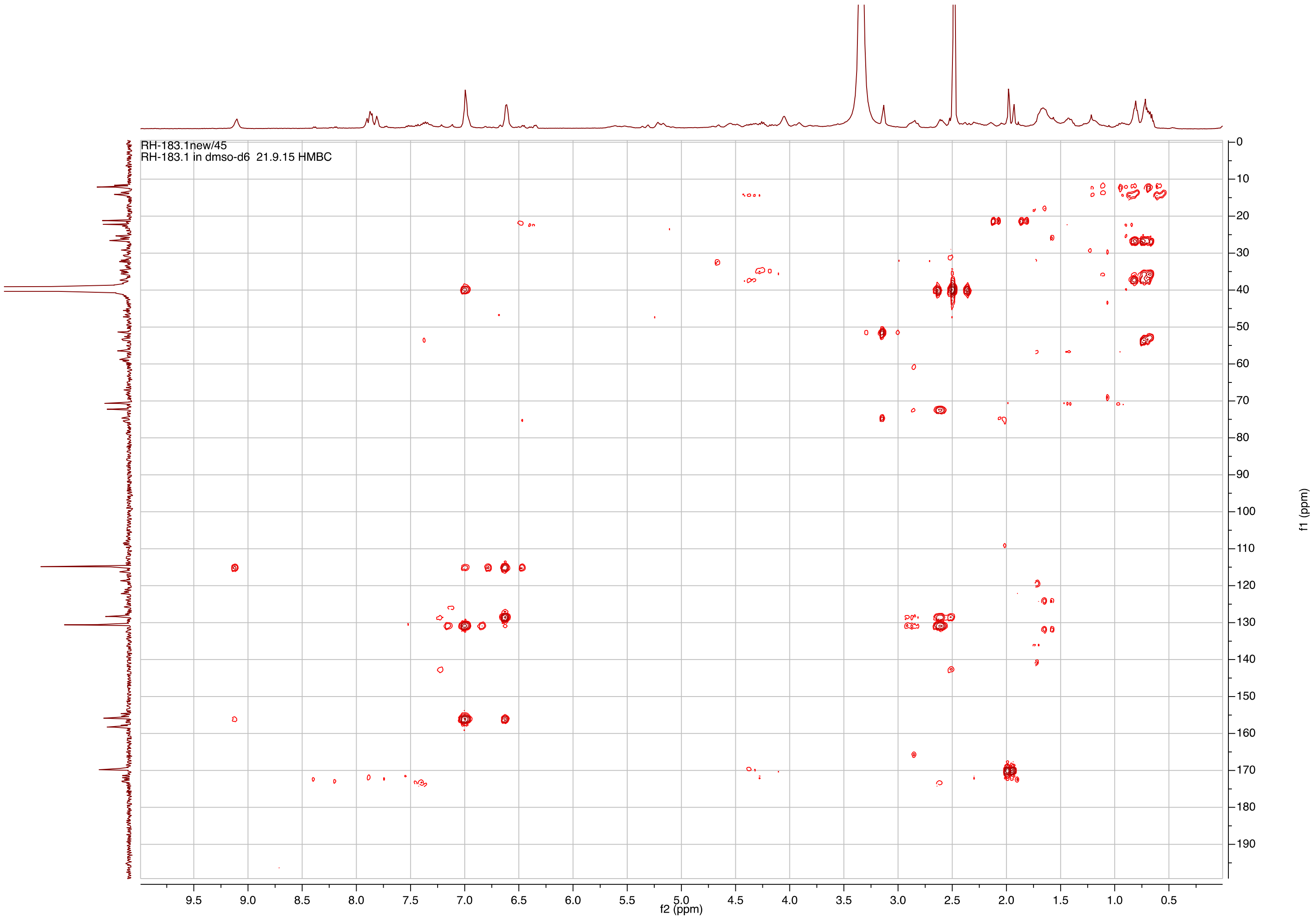

S16. COSY Spectrum of Aeruginosin TR642 (**2**) in DMSO-d_6

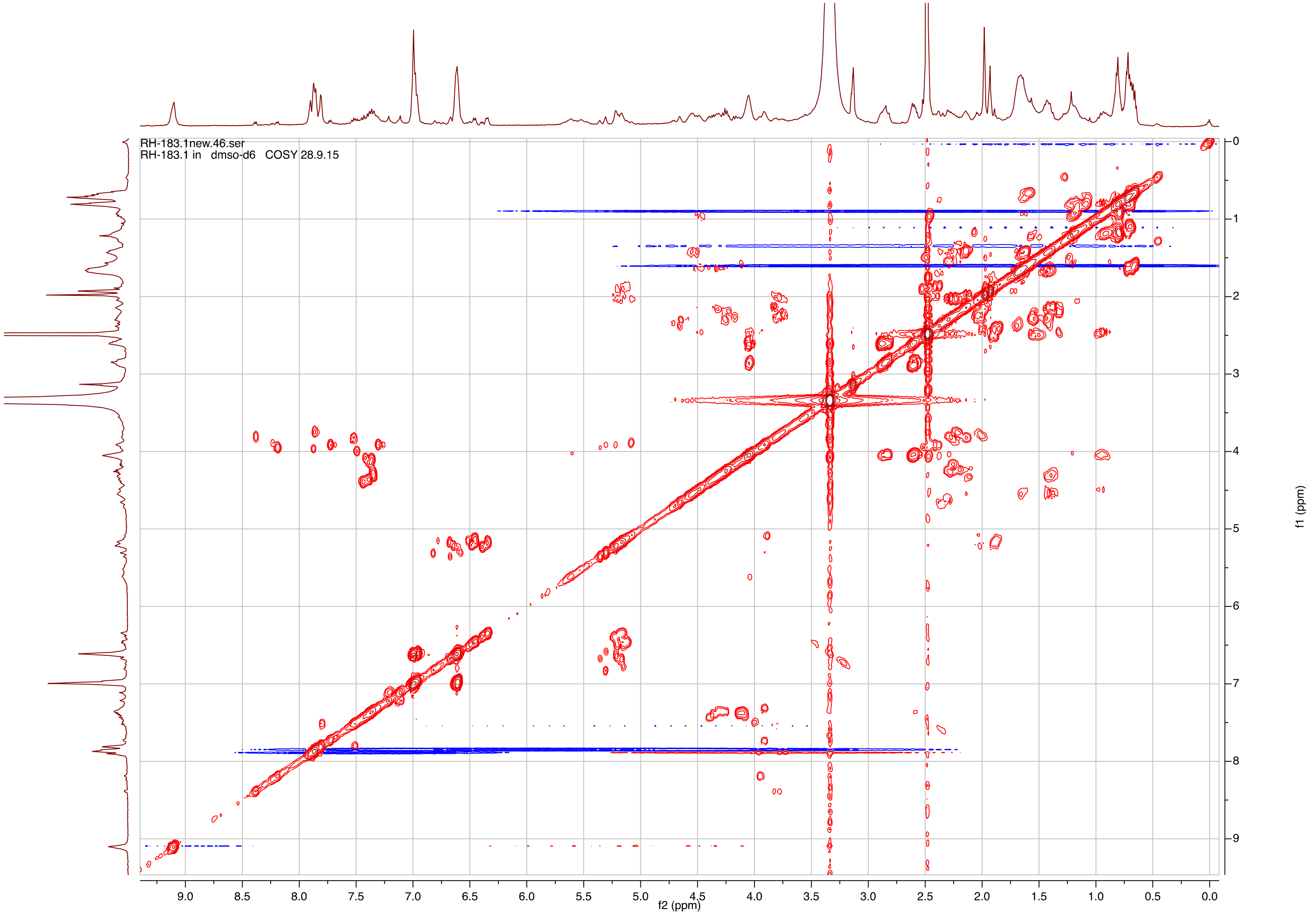

S17. TOCSY Spectrum of Aeruginosin TR642 (**2**) in DMSO-d_6

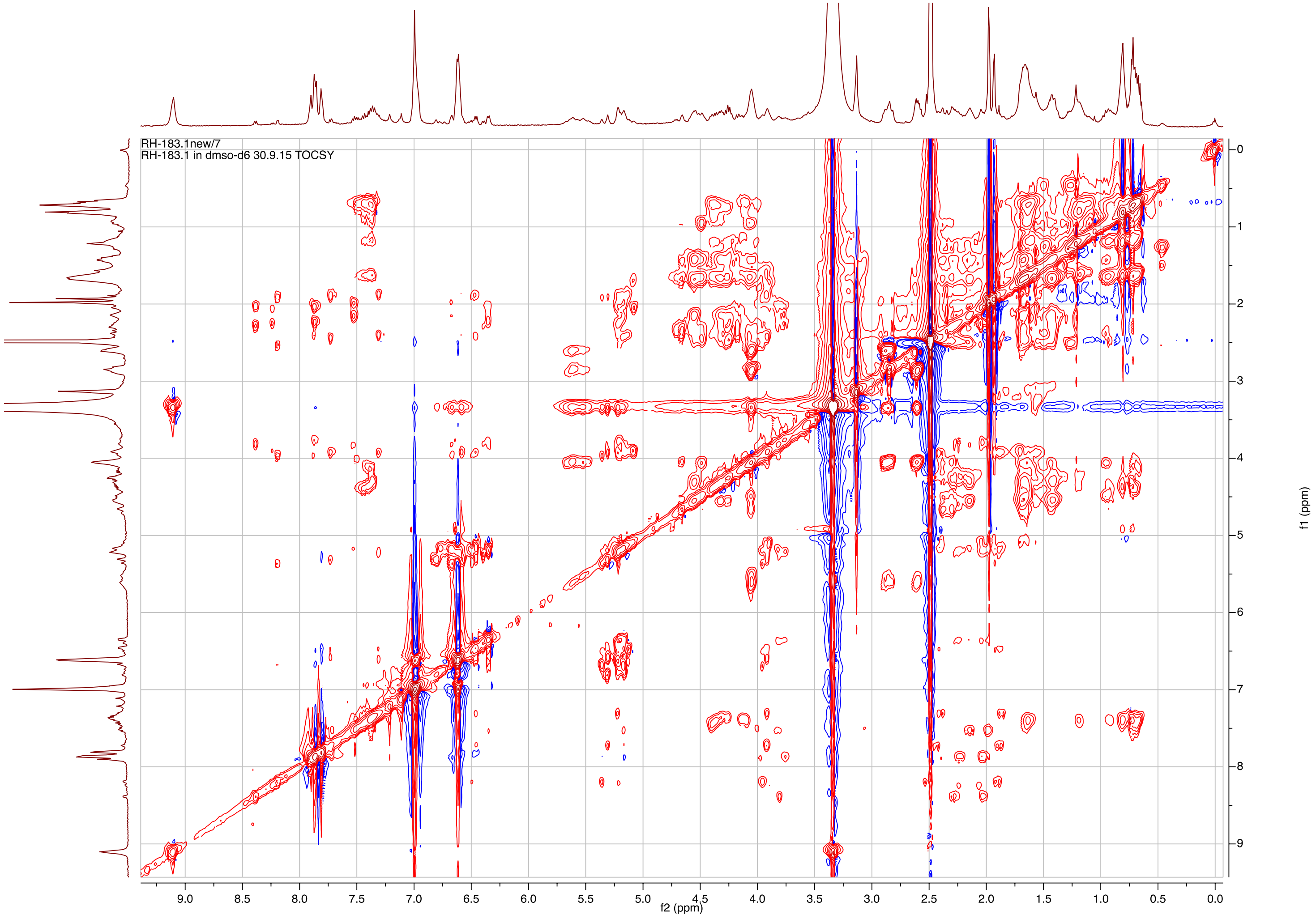

S18. ROESY Spectrum of Aeruginosin TR642 (**2**) in DMSO-d_6 (NOE correlation)

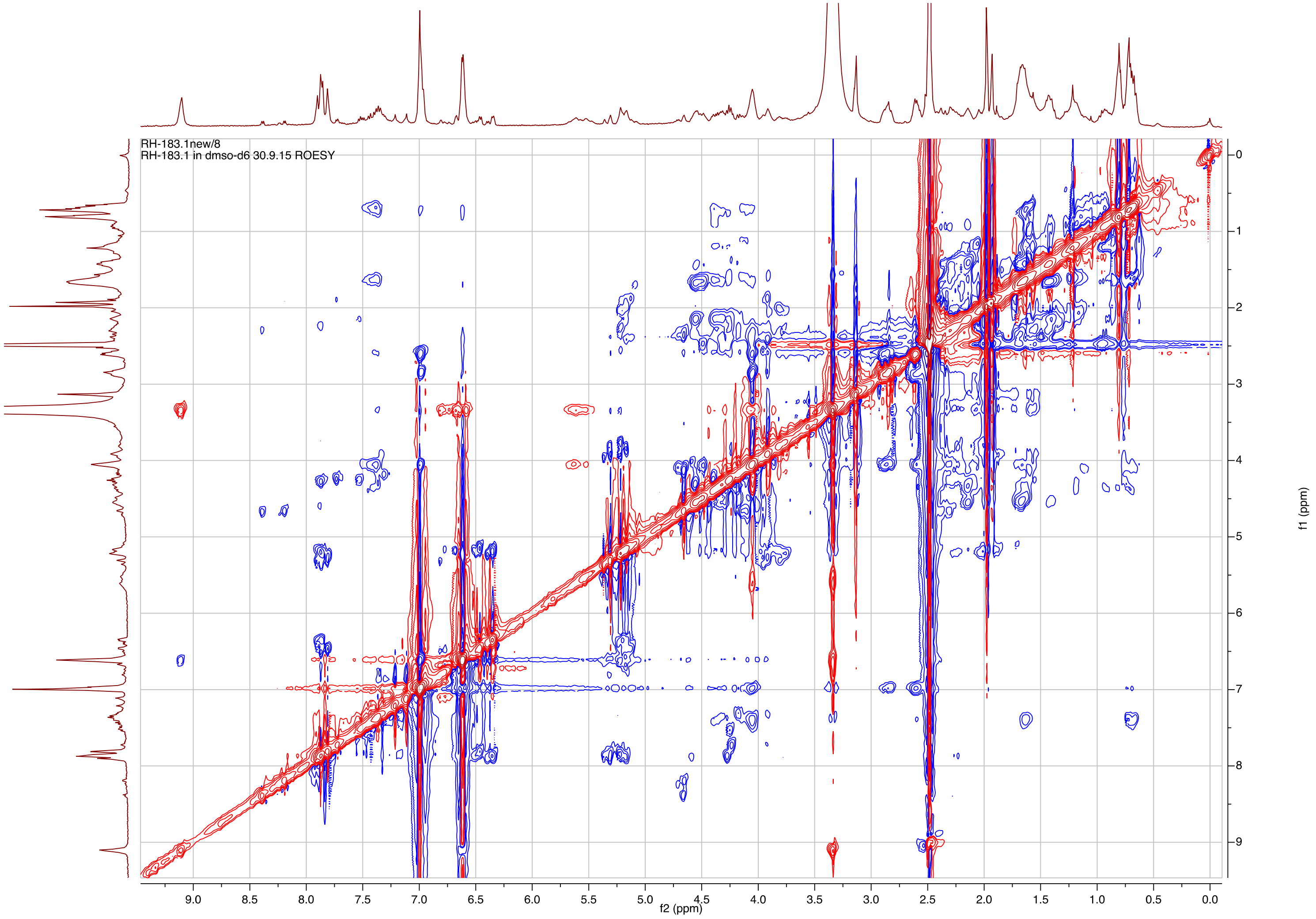

S19. HR ESI MS data of Aeruginosin TR642 (**2**)

Elemental Composition Report **Page 1**

Single Mass Analysis
Tolerance = 20.0 PPM / DBE: min = -1.5, max = 50.0
Element prediction: Off
Number of isotope peaks used for i-FIT = 3

Monoisotopic Mass, Even Electron Ions
323 formula(e) evaluated with 18 results within limits (up to 5 closest results for each mass)
Elements Used:
C: 28-35 H: 40-60 N: 0-10 O: 0-15 Na: 0-1
RH-183.1
CARMELI1126 34 (1.507) Cm (32:34)

Rawan Hasan

1: TOF MS ES+
2.34e+005

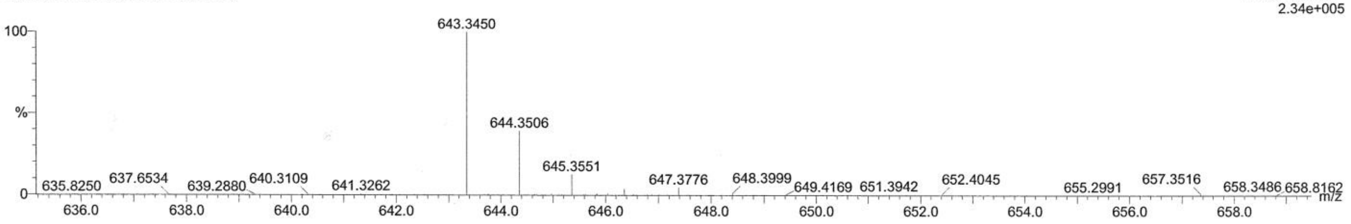

Minimum:				-1.5			
Maximum:		5.0	20.0	50.0			
Mass	Calc. Mass	mDa	PPM	DBE	i-FIT	i-FIT (Norm)	Formula
643.3450	643.3455	-0.5	-0.8	12.5	268.3	1.7	C32 H47 N6 O8
	643.3445	0.5	0.8	14.5	269.3	2.8	C31 H44 N10 O4 Na
	643.3458	-0.8	-1.2	8.5	267.2	0.7	C34 H52 O10 Na
	643.3442	0.8	1.2	7.5	268.3	1.7	C31 H51 N2 O12
	643.3431	1.9	3.0	9.5	269.3	2.7	C30 H48 N6 O8 Na
